# Differential expression and localization of expansins in *Arabidopsis* shoots: implications for cell wall dynamics and drought tolerance

**DOI:** 10.3389/fpls.2025.1546819

**Published:** 2025-02-10

**Authors:** Darina Balkova, Katerina Mala, Jan Hejatko, Klara Panzarova, Lamis Abdelhakim, Barbora Pleskacova, Marketa Samalova

**Affiliations:** ^1^ Department of Experimental Biology, Faculty of Science, Masaryk University, Brno, Czechia; ^2^ CEITEC – Central European Institute of Technology, Masaryk University, Brno, Czechia; ^3^ National Centre for Biomolecular Research, Faculty of Science, Masaryk University, Brno, Czechia; ^4^ PSI (Photon Systems Instruments) Ltd., Drasov, Czechia

**Keywords:** cell wall, EXPA, *Arabidopsis*, highthroughput phenotyping, abiotic stress

## Abstract

Expansins are cell wall-modifying proteins implicated in plant growth and stress responses. In this study, we explored the differential localization of expansins in *Arabidopsis thaliana* shoots, with a focus on *EXPA1, EXPA10, EXPA14*, and *EXPA15* utilizing *pEXPA::EXPA* translational fusion lines. Employing the chemically inducible system pOp6/LhGR for *EXPA1* overexpression and high-throughput automatic phenotyping we evaluated the drought response and photosynthetic efficiency under stress conditions. We observed distinct expression patterns of expansins, with *EXPA1* primarily localized in stomatal guard cells, while *EXPA10* and *EXPA15* showed strong cell wall (CW) localization in epidermal and other tissues. Overexpression of *EXPA1* resulted in pronounced changes in CW-related gene expression, particularly during early stages of induction, including the upregulation of other expansins and CW-modifying enzymes. The induced *EXPA1* line also displayed significant morphological changes in shoots, including smaller plant size, delayed senescence, and structural alterations in vascular tissues. Additionally, *EXPA1* overexpression conferred drought tolerance, as evidenced by enhanced photosynthetic efficiency (F_v_/F_M_), and low steady-state non-photochemical quenching (NPQ) values under drought stress. These findings highlight the critical role of *EXPA1* in regulating plant growth, development, and stress response, with potential applications in improving drought tolerance in crops.

## Introduction

The ability of plants to endure environmental stress is a critical determinant of their growth and development. Among various stressors, drought is one of the most detrimental to plant health, as it directly restricts water availability and impacts numerous physiological and metabolic processes (Salehi-Lisar and Bakhshayeshan-Agdam, 2016). In response to such challenges, plants have evolved intricate adaptive mechanisms (Huang et al., 2012), with the cell wall (CW) playing a pivotal role in responding to environmental stimuli (Cosgrove, 2000a). The dynamic nature of the CW is largely regulated by expansins, which are key agents in CW remodeling. This gene family is classified into four subgroups: *α-EXPANSIN (EXPA), β-EXPANSIN (EXPB), EXPANSIN-like A (EXLA)*, and *EXPANSIN-like B (EXLB)*, each contributing uniquely to various aspects of plant development (Lee et al., 2001; Sampedro and Cosgrove, 2005; Cosgrove, 2015; Samalova et al., 2022). Expansins, identified as non-enzymatic CW proteins, facilitate “cell wall loosening” by disrupting the non-covalent hydrogen bonds between cellulose microfibrils and hemicellulose xyloglucans, allowing for the turgor-driven cell expansion (Cosgrove, 2000b; Cosgrove, 2005; Cosgrove, 2016; Cosgrove, 2024). Through this mechanism, expansins modulate the structural flexibility of the CW which is essential for maintaining cell turgor and promoting growth under both normal conditions and in response to abiotic and biotic stressors (Lee et al., 2001; Cosgrove, 2005; Gao et al., 2010). The expression of expansin genes in *Arabidopsis thaliana* is regulated by various internal and external factors, such as phytohormones, developmental stages, and environmental stressors (Samalova et al., 2022). These genes are predominantly expressed in rapidly elongating cells, the root and shoot apical meristems, leaf primordia, and young stems; however, the expression patterns of individual expansin genes vary according to their specific functions (Cho and Cosgrove, 2000; 2002; McQueen-Mason and Cosgrove, 1995; Samalova et al., 2024).

In response to drought stress, plants undergo various physiological, biochemical, and molecular changes, including stomatal closure, accumulation of osmolytes, and activation of stress-responsive genes (Shao et al., 2008a, b). Numerous studies have suggested that modifying expansin expression may facilitate these critical physiological and molecular responses, potentially participating in stress-signaling pathways that enhance plant resilience under water-limited conditions (Chen et al., 2016, 2018; Dai et al., 2012; Guo et al., 2011; Hao et al., 2017; Kwon et al., 2008; Li et al., 2011; Liu et al., 2019; Lu et al., 2013; Marowa et al., 2020; Narayan et al., 2019, 2021; Wu et al., 2001; Zhang et al., 2021). Most of these studies link increased plant drought tolerance due to overexpression of expansins with enhanced root growth and improved root architecture, thereby potentially increasing water uptake and nutrient acquisition (Cho and Cosgrove, 2002; Guo et al., 2011; Lee et al., 2003; Wu et al., 2001). Interestingly, a recent study using a machine learning-driven meta-analysis of all available stress-related transcriptomic data identified expansins as core stress genes in roots (Sanchez-Munoz et al., 2024).

However, the impact of drought extends beyond root growth, affecting the entire plant in a complex manner, including shoot development and processes such as photosynthesis and gas exchange (Cho and Cosgrove, 2000; Flexas et al., 2004; Chaves et al., 2009). For instance, Dai et al. (2012) indicated that the expansin gene *RhEXPA4* plays a regulatory role in dehydration tolerance during the expansion of rose *(Rosa hybrida)* petals. Furthermore, transgenic *Arabidopsis* plants overexpressing *RhEXPA4* exhibited enhanced drought tolerance and improved recovery following a drought period compared to wild-type (WT) plants. Similarly, in tobacco (*Nicotiana tabacum*), the overexpression of the wheat (*Triticum aestivum*) *TaEXPB23* gene enabled transgenic plants to retain water for a longer period under drought stress than WT plants, as indicated by higher maximum fluorescence in dark adapted state (QY_max_) and steady-state Photosystem II quantum yield (ΦPSII) values and reduced electrolyte leakage (Li et al., 2011). Likewise, overexpression of *EaEXPA1* from WT sugarcane (*Erianthus arundinaceus*) in the Co 86032 cultivar resulted in higher relative water content, improved membrane thermostability, increased chlorophyll content, enhanced quantum yield and gas exchange under drought stress, compared to WT plants (Narayan et al., 2021). The observed increase in photosynthetic capacity during drought stress due to expansin overexpression may be attributed to enhanced growth and leaf formation, resulting in an expanded photosynthetically active area and improved light capture under stress conditions (Chen et al., 2018; Marowa et al., 2020; Zhang et al., 2021). Moreover, research indicates that expansin overexpression may influence stomatal dynamics and accelerate stomatal movements (Wei et al., 2011; Zhang et al., 2011) and reduce stomatal density under drought conditions (Lu et al., 2013). This suggests a role of expansins in regulating stomatal movements - an essential process for minimizing water via transpiration during drought (Farooq et al., 2009).

Expansin upregulation under drought stress is also associated with the activation of signaling pathways and stress response genes involved in osmolyte accumulation, antioxidant defense, and hormone signaling (Shao et al., 2008a; Chen et al., 2016, 2018). For example, overexpression of wheat *TaEXPA2* in tobacco enhanced drought tolerance by improving the water management and promoting the accumulation of osmotically active substances, such as proline. Transgenic plants also showed higher antioxidant levels and reduced reactive oxygen species (ROS) accumulation (Chen et al., 2016). Similarly, the ability to scavenge ROS was significantly improved in transgenic *Arabidopsis* plants overexpressing *Ammopiptanthus nanus AnEXPA1* and *AnEXPA2* (Liu et al., 2019). In cotton, overexpression of *GhEXLB2* improved drought tolerance during critical stages such as germination, early growth, and flowering, reduced ROS accumulation, and increased antioxidant enzyme activity. Transgenic plants also displayed higher chlorophyll content, osmoprotective soluble sugars, and water-use efficiency compared to WT plants (Zhang et al., 2021).

Based on these findings, it might be expected that knock-out mutants of individual expansin genes would exhibit reduced biomass growth and increased sensitivity to abiotic stress. However, the literature suggests that expansin mutants often show no significant phenotypic changes, likely due to functional redundancy within the expansin gene family, a phenomenon observed in other gene families as well. Thus, simultaneous mutations in multiple expansin genes may be necessary to produce significant phenotypic changes (Cosgrove et al., 2002; Schipper et al., 2002; Li et al., 2003). Nevertheless, the precise molecular mechanisms by which expansins contribute to drought tolerance remain an active area of research. The interplay between expansins and other drought-responsive pathways highlights the complexity of expansin*-*mediated stress responses in plants (Cutler et al., 2010).

In this study we describe the expression of several hormone-responsive expansins in the shoots of *Arabidopsis thaliana*, explore genome-wide changes associated with *AtEXPA1* overexpression (*EXPA1* OE), and investigate the responses of overexpression and genetically engineered multiple knocked-out lines under abiotic stress conditions, focusing on drought stress. Understanding these mechanisms is essential not only for advancing knowledge of plant stress biology but also for developing crops with enhanced resilience to changing environments, thereby contributing to the sustainability of agriculture in the face of global climate change.

## Materials and methods

### Plant cultivation conditions and dexamethasone induction *in vitro*

Seedlings of *Arabidopsis thaliana* (ecotype Col-0) were grown *in vitro* on standard Murashige and Skoog (MS) medium (Murashige and Skoog, 1962) supplemented with 1.5% (w/v) sucrose and 0.8% (w/v) plant agar (Duchefa), with the pH adjusted to 5.8. For osmotic stress experiments various concentrations of NaCl (75, 150, 225 mM) were added to the medium, along with 20 *μ*M Dex for induction, seedlings were germinated and grown on the plates for 7 days. The plants were cultivated in growth chambers under long-day conditions (16 h light/8 h dark) at 21°C during the light phase and 19°C during the dark phase (21°C/19°C).

### Growth conditions and high-throughput phenotyping analysis of soil-grown plants

Plants were cultivated as described in Awlia et al., 2016 and Flutsch et al., 2020, with the following modifications. Seeds were sown on soil (Substrate 2, Klasmann-Deilmann GmbH, Germany), stratified at 4°C for 3 days in the dark, and transferred to a climate-controlled chamber (FytoScope FS-WI, PSI, Drasov, Czech Republic) under 12h/12h, 21°C/19°C light/dark photoperiod with a relative humidity of 60% (for control condition experiment) and 45% (for drought stress experiment) and an irradiance of 120 µmol m^−2^ s^−1^ (cool-white and far-red LED).

*Control condition experiment:* Seeds of wild-type (WT) and Dex-inducible *pRPS5A>GR>EXPA1* overexpressing (*EXPA1 OE*) lines 5-4 and 8-4 (Samalova et al., 2024) were sown in pots. Seven days after stratification (DAS), seedlings of similar size were transplanted into single pots prepared the day before with 95 g of sieved soil and watered up to the maximum soil-water holding capacity either with water or Dex solution (20 *μ*M). Plants were cultivated in the climate-controlled chamber as described above. At 18 DAS plants were placed randomly in trays (10 pots per tray) and into the PlantScreen™ Compact System (PSI, Czech Republic) installed in controlled environment (FytoScope FS_WI, PSI, Czech Republic) with the same conditions as described above. Plants were watered manually every 2-3 days with 10 ml of 20 *μ*M Dex solution as described in Samalova et al., 2019. Automated phenotyping started at 18 DAS and continued until 46 DAS. Plants were imaged daily using Red Green Blue (RGB) imaging. The PlantScreen™ Compact System facilitated the daily transport of trays for phenotypic analyses on conveyor belts from the dark/light acclimation chamber to the light-isolated imaging cabinets and the weighing and watering station, where plants were automatically weighed and watered daily to maintain the soil at a relative water content (RWC) of 60% field capacity.

*Drought stress experiment:* WT, *EXPA1 OE* line (8-4), and triple mutant (*expa1,10,14-1 and -2* generated in this study) seeds were planted in soil. The *EXPA1 OE* line and WT were treated with or without Dex. Plants were cultivated as described above (control experiment) with the following modifications. Drought experiment was designed according to Clauw et al., 2015 with first drought phase from 10 DAS until 23 DAS followed by recovery phase for 6 days (from 24 to 29 DAS) and initiation of second drought phase from 30 DAS till 36 DAS. Seven DAS seedlings of similar size were transplanted into single pots prepared the day before with 65 g of sieved soil and watered regularly up 44% RWC (control levels; refers to 2.2 g of water per 1 g of dry soil) with water or with Dex solution (20 *μ*M). At 10 DAS progressive drought stress was induced by watering half of the plants only up to 22% RWC (refers to 2.2 g of water per 1g of dry soil) with water or with Dex solution. Plants were cultivated in the climate-controlled chamber and 10 DAS were placed randomly in trays (10 pots per tray) and into the PlantScreen™ Compact System (PSI, Czech Republic) installed in controlled environment (FytoScope FS_WI, PSI, Drasov, Czech Republic). Automated phenotyping started 10 DAS and continued daily until 36 DAS. The photoperiod was changed to 16 h light/8 h dark, and humidity was kept at 45%. Successively, plants were automatically phenotyped using kinetic chlorophyll fluorescence imaging and RGB imaging, and automated watering and weighing in the listed order. The changes in the photosynthesis-related parameters were measured at different photon irradiances using the light curve protocol as described in Awlia et al., 2016. Briefly, a 5s flash of light was applied to measure the minimum fluorescence, followed by a saturation pulse of 800 ms (with an irradiance of 1200 μmol m^-2^ s^-1^) to determine the maximum fluorescence in the dark-adapted state. Next, 60 s intervals of cool-white actinic light at 95, 210, 320, 440 μmol m^-2^ s^-1^ corresponding to L1, L2, L3, and L4, respectively, were applied. A saturation pulse was applied at the end of the period of actinic light to acquire the maximal fluorescence in the light- adapted state. The chlorophyll fluorescence (Chl) signal measured just before the saturation pulse was taken as the steady-state fluorescence value in the light-adapted state. Using the automatic timing function of PlantScreen™ Scheduler (PSI, Drasov, Czech Republic), the phenotyping protocol was programmed to start always at the same time of the diurnal cycle (after 2 h of illumination in the climate-controlled growth chamber). A total of 26-28 plants per treatment and line were used.

Image segmentation and automatic raw data processing were performed using the PlantScreen™ Analyzer software (v3.3.10.7, PSI, Drasov, Czech Republic). The parameters measured and calculated in this study, based on spectral values, are listed in **Supplementary Table S1**.

### CRISPR/Cas9 cloning and knock-out plant preparation

The CRISPR/Cas9 constructs for multiplex mutagenesis were prepared at the Vienna BioCenter Core Facilities GmbH (Dr. Bohr-Gasse 3, 1030 Vienna) by Dr. Vera Schoft. GreenGate entry vectors were combined into a single plant transformation vector through a GoldenGate reaction, as described by Richter et al. (2018), with the modification of using a yellow fluorescent protein seed coat marker (Alli-YFP) for the selection of transgenic lines. Briefly, the final construct comprised the ubiquitin4-2 promoter from *Petroselinum crispum*, an *Arabidopsis* codon optimized Cas9, the pea3A terminator from *Pisum sativum*, and three guide RNA (gRNA) modules, each consisting of the *Arabidopsis* U6-26 promoter, a guide sequence, and the gRNA scaffold (Richter et al., 2018). Additionally, a simple construct carrying a single gRNA for *EXPA10* was also prepared.

The following gRNA sequences targeting the *α-EXPANSIN* family members were selected using the Broad institute tool ( *www.broadinstitute.org/rnai/public/analysis-tools/sgrna-design* ):

*EXPA10* (At1g26770): GTGTTAACAACCTGCACAT (exon 1),

*EXPA14* (At5g56320): AGCGTGGATGGTTACAGTAG (exon 1),

*EXPA15* (At2g03090): TACGGGAACCTCTACAGTCA (exon 2).

The constructs were transformed into *Agrobacterium tumefaciens* GV3101 pSOUP+ cells and subsequently used for *Arabidopsis* transformation via the floral dip method (Clough and Bent, 1998). Both wild-type (WT) and the *expa1-2* CRISPR/Cas9 line generated by Ramakrishna et al. (2019) were transformed to select for single and multiple expansin mutants. Transgenic T1 generation seeds exhibiting fluorescence were selected using an Olympus SZX16 fluorescence stereomicroscope and a GentleGrab tweezer (Labdeers s.r.o., Czech Republic). These seeds were propagated to the T2 generation, and only non-fluorescent lines that segregated out the T-DNA were sequenced for putative insertions and deletions (indels). Sequencing PCR reactions using isolated genomic DNA (gDNA) as a template were performed with following primers: *EXPA10-F* CAAGTACGTAACTACTTTCTCC and *EXPA10-R* CATTGTGCCGGAAGCATCACC, *EXPA14-F* CTCTTCTTCCTCATTTTCTTCC and *EXPA14-R* GTTTCCGTAACCACACGCGCC, *EXPA15-F* CTCATCCTACCTTCTATGGTGG and *EXPA15-R* GTTAGGAGGACAGAAATTGGTGG.

T2 progeny of 4 independent transgenic lines were tested for *expa10*, 8 lines for the *expa1expa10* double mutant, 42 lines for the *expa10expa14expa15* triple mutant and 85 lines for the *expa1expa10expa14expa15* quadruple mutant. A summary of the mutant lines used in this study is provided in **Table 1**. Despite our efforts we were unable to identify any viable mutants in *EXPA15*. Insertions in the targeted sequence of *EXPA10* created a stop codon after 46 amino acids (aa). In the targeted sequence of *EXPA14*, insertions created a stop codon after 25 aa and the deletion after 65 aa or it interfered with intron excision.

**Table 1:** Mutations identified in selected *EXPA* genes.

Line name and number	Mutation		
	*expa1*	*expa10*	*expa14*
*expa1-2*	deletion	–	–
*expa10-1* (8-10)	–	insertion (A)	–
*expa10-2* (10-1)	–	insertion (A)	–
*expa1,10-1* (2-1)	deletion	insertion (T)	–
*expa1,10-2* (3-5)	deletion	insertion (T)	–
*expa10,14-1* (11-1)	–	insertion (T)	insertion (A)
*expa10,14-2* (11-2)	–	insertion (A)	insertion (A)
*expa1,10,14-1* (4-6)	deletion	insertion (T)	insertion (G)
*expa1,10,14-2* (14-6)	deletion	insertion (T)	deletion (A)

### RNAseq and differential expression analysis

RNA seq libraries were prepared from 10 μg of total RNA isolated from shoots of 7-day old *Arabidopsis* seedlings of WT and *EXPA1 OE* line 8-4 (Samalova et al., 2024) using the RNeasy Plant Mini Kit (QIAGEN). Seedlings were grown on MS medium +/- Dex for 7 days or induced with Dex for 3 hours, with four biological replicas for each treatment. Libraries were constructed using the NEBNext Ultra II Directional RNA Library Prep Kit for Illumina, incorporating polyA selection, and sequenced with 75 base paired-end reads. Unique Molecular Identifiers (UMIs) were included in the samples (6 bp long) to detect PCR duplicates. Raw sequencing reads were quality-checked using FastQC, MinION, and Swan, pre-processed with Trimmomatic, and aligned to the *Arabidopsis thaliana* reference genome (TAIR10-31) using the STAR aligner (v2.7.3a) with gene annotation. Gene counts were obtained using RSEM, and differential expression (DE) analysis was conducted using DESeq2. Differentially expressed genes were identified based on an adjusted p-value of < 0.05 and a log2FoldChange threshold of ±1. Gene ontology (GO) annotations and analyses were performed using GOrilla (Eden et al., 2009). The data discussed in this publication have been deposited in NCBI’s Gene Expression Omnibus (Edgar et al., 2002) and are accessible through GEO Series accession number GSE286232 (https://www.ncbi.nlm.nih.gov/geo/query/acc.cgi?acc=GSE286232).

### Confocal laser scanning microscopy

Localization of *EXPA:mCherry* fusions was performed using Zeiss LSM 880 confocal laser scanning microscope. mCherry fluorescence was detected within the 580-650 nm range using a 561-nm HeNe laser for excitation, while eGFP fluorescence was detected within the 490-550 nm range using a 488-nm Argon laser line. Z-stack images were acquired using a 40×/1.2 water-corrected C-Apochromat objective and are presented as maximum intensity projections.

### Quantitative analysis of leaf epidermal cells

Leaf epidermal images were captured using a 3D microscope VHX-7000 Series (Keyence) at a resolution of 1600x1200 pixels. Cell count and areas were analyzed in ImageJ (version 1.54k) using the PaCeQuant plugin (Poeschl et al., 2020) integrated with MiToBo (version 2.3.1). Automated segmentation and quantification of cell shape parameters were performed. Pixel size (0.216 µm/pixel) was calibrated from a 50 µm scale bar corresponding to 231.5 pixels. Areas in pixels² were converted to µm² using Area (µm^2^) = Area (pixels^2^) × (0.216)^2^. Stomatal aperture width was determined manually by using the line tool in ImageJ, while stomatal density was calculated by counting stomata within a defined area (≈0.0897 mm²) and expressed as number of stomata per mm².

### Statistical analysis

Statistical analyses were performed using ANOVA followed by *post hoc* Tukey HSD (Unequal N HSD test) in STATISTICA (TIBCO Software Inc.; version 14.0.0.15). Data were tested for normality using the Shapiro-Wilk test and for variance homogeneity using Levene’s test prior to analysis. Results are presented as means ± standard deviations (S.D.), with statistically significant differences (p < 0.05) indicated by differing lowercase letters. For the phenomics data, statistical analyses were performed using R studio software (Version 2023.12.1). Outlier detection was done using “rstatix” package via identify outliers’ function and data imputation was done afterward using “missforest” package in the data pre-process. Data were checked for variance homogeneity with Levene`s test using “car” package and normal distribution using Shapiro-test before statistical analysis. The data were analyzed for each experiment separately focusing on comparing genotypes and treatments across timepoints (DAS). Means were compared with a *post-hoc* pairwise comparison procedure using the Dunn test and adjusted with Benjamini-Hochberg correction, and the significant difference was indicated by different small letters using “multcompView”. To investigate significant differences among lines, for non-normal data analysis, Kruskal-Wallis test was used, with *post-hoc* testing (Dunn test) to identify pairwise differences. For the drought stress experiment, the Mann-Whitney U test was applied to assess treatment-specific differences. The level of significance of each factor is indicated as *p < 0.05, ** p < 0.01, *** p < 0.001, **** p < 0.0001, and ns indicates non-significant differences. By combining all traits, Random Forest model was used to find traits of interest across all time points using “randomForest” package and presented as variable importance where important 20 traits were selected and ranked. Variables were ranked based on their importance, where higher values indicated more contributions to prediction accuracy. Moreover, k-means clustering in a two-dimensional obtained through principal component analysis (PCA) was used for group mean, to discriminate between the lines under each treatment separately by using “factroextra”, “mixOmics”, “ggfortify”, “PCAtools” packages.

### Accession numbers

Sequence data associated with this study can be found in the GenBank/EMBL data libraries under the following accession numbers: *EXPA1* (*At1g69530*), *EXPA10* (*At1g26770*), *EXPA14* (*At5g56320*), and *EXPA15* (*At2g03090*).

## Results

### Expansins exhibit differential localization in the cell walls of *Arabidopsis* shoot

Our previous study demonstrated the localization of expansins within the CW of *Arabidopsis thaliana* roots using translational fusions of EXPA1, EXPA10, EXPA14, and EXPA15 with the red fluorescent protein mCherry, under the control of their native promoters (Samalova et al., 2024). In this study, we employed the same *pEXPA::EXPA:mCherry* lines to investigate expansin localization in *Arabidopsis* shoots.

**Figure 1:**
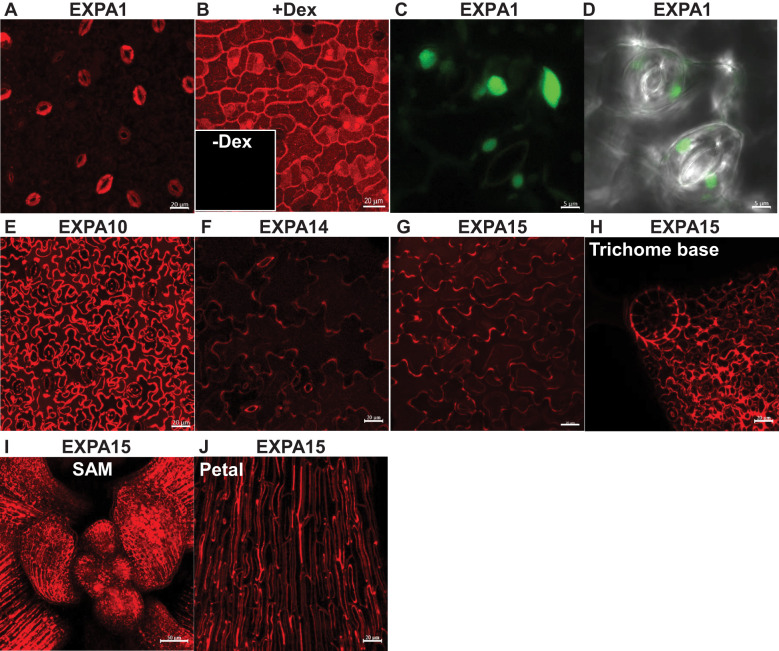
Expansin promoter activity and localization in *Arabidopsis* shoots. Z-stack projections of **(A)** *pEXPA1::EXPA1:mCherry* fusion showing localization in the stomatal guard cells and the epidermis, **(B)** *pRPS5A>GR>EXPA1:mCherry* (line 1-3) plants grown on MS media with dexamethasone (+Dex) showing localization in the CWs of all cells, as well as within the vacuoles of developing stomata; insert without Dex (-Dex). Z-stack projections of **(C)** *pEXPA1::nls:3xGFP* illustrating a similar pattern of *EXPA1* expression, with GFP signal visible in the nuclei of stomatal guard cells (round) and epidermal cells (ellipsoid) and **(D)** transmitted-light micrograph of a single optical section. Z-stack projections of **(E)** *pEXPA10::EXPA10:mCherry* fusion localized in the CW of epidermal and underlying parenchymal cells, **(F)** *pEXPA14::EXPA14:mCherry* showing localization in the CW of epidermal cells and autofluorescence of the thick inner CW of stomata pores, **(G)** *pEXPA15::EXPA15:mCherry* localized in the CW of epidermal cells, **(H)** a trichome base, **(I)** shoot apical meristem (SAM) and **(J)** petal leaves. The plants shown were 7-14 days old, except for the 5-week-old plant in **(J)**. Scale bars correspond to 20 μm, except for **(C, D, I)**, which correspond to 5 μm and 50 μm, respectively.

Notably, *EXPA1* exhibited the highest expression in stomatal guard cells (**Figure 1A**), with localization observed not only in the CW but also within vacuoles. However, a weak signal resembling CW localization in the epidermis was also detectable. Furthermore, when overexpressed in dexamethasone (Dex)-inducible *pRPS5A>GR>EXPA1:mCherry* plants (Samalova et al., 2024), strong mCherry fluorescence was detectable in the CWs of all cells, as well as within the vacuoles of developing stomata (**Figure 1B**). The prevalence of shoot *EXPA1* expression in stomata was corroborated using an independent transcriptional fusion line, *pEXPA1::nls:3xGFP* (Ramakrishna et al., 2019), which exhibited GFP fluorescence within the nuclei of guard cells and epidermis (**Figures 1C, D**).

Compared to EXPA1, EXPA10 provided a substantially stronger fluorescence signal, predominantly localized to the CW of epidermal cells and likely the underlying parenchymal cells (**Figure 1E**). In contrast, *EXPA14* showed weaker promoter activity, similar to *EXPA1*, though it displayed a characteristic CW labelling pattern in epidermal cells. Due to the relatively high detector gain, autofluorescence of the thick inner CW surrounding the stomatal pore was also visible (**Figure 1F**). *EXPA 15* expression was robust across the leaf epidermis and various specialized cell types, including those at the trichome base, the shoot apical meristem (SAM) and petal leaves (**Figures 1G–J**), implying a crucial role in plant growth and development.

In summary, each of the tested expansins exhibits distinct expression and localization patterns in *Arabidopsis* shoots, including leaves and cotyledons.

### *EXPA1* overexpression increases stomata density and aperture width

Previously, we generated Dex-inducible *pRPS5A>GR>EXPA1* (*EXPA1 OE*) lines (Samalova et al., 2024). To complete the genetic toolbox, here using CRISPR/Cas9 technology, we created single (*expa10*), double (*expa1,10* and *expa10,14*) and triple (*expa1,10,14*) expansin knock-out mutants. To evaluate their potential resistance to osmotic stress, we compared these mutants, along with WT and *EXPA OE* line 8-4 by growing them on MS media supplemented with various concentrations of NaCl. The measured phenotypes included germination rate, root length, and fresh shoot weight (**Supplementary Figure S1**). However, no significant differences were observed across the lines, except for some variation in germination, suggesting limited osmotic stress tolerance under *in vitro* conditions.

**Figure 2:**
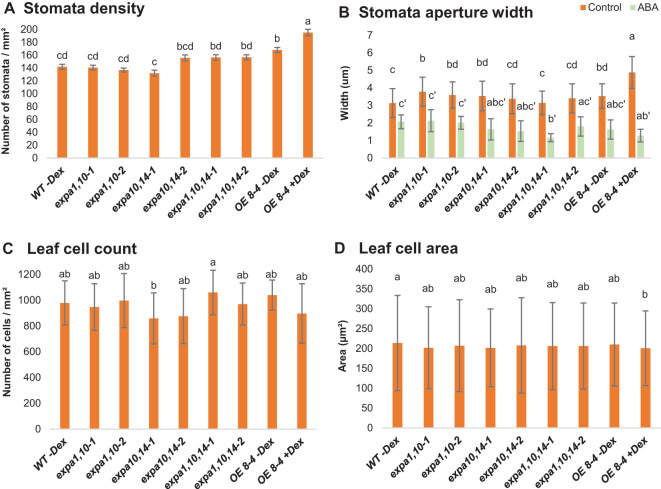
Stomata and leaf cell characterization. Graphs represent **(A)** stomata density, **(B)** stomata aperture width, **(C)** number of cells per square mm (mm^2^) and **(D)** size of cells (μm^2^) in WT, *expansin* mutants (*expa1,10-1* and *-2*, *expa10,14-1* and *-2*, *expa1,10,14-1* and *-2*) and *EXPA1 OE* line (8-4; -Dex and +Dex) after 4 weeks of soil growth. Rosette leaves were excised, immersed in water supplemented with 10 mM ABA for 30 min (where indicated), illuminated with white light for 30 min, and subsequently photographed. Error bars represent S.D. with 4-6 plants per treatment and line analyzed. Significant differences among genotypes (at each treatment) were assessed using the *post hoc* Tukey HSD test (Unequal N HSD). Different lowercase letters above the barplot indicate statistically significant differences (p < 0.05).

To further investigate the role of assayed EXPAs in stomata and their potential contribution to drought resistance, we conducted experiments on soil-grown WT, double, and triple expansin mutants, alongside the *EXPA1 OE* line (8-4) with and without Dex induction. The plants were grown for four weeks before excising rosette leaves for analysis. The leaves were illuminated with white light for 30 minutes, after which stomata density (**Figure 2A**) and stomata aperture width (**Figure 2B**) were measured. Our results revealed that the *EXPA1 OE* line had the highest stomata density compared to all other lines, with the stomatal pores being significantly more open. This suggests that *EXPA1* overexpression (OE) enhances stomata formation and aperture size. Interestingly, treatment with abscisic acid (ABA) had no differential effect on stomata closure across the genotypes tested, indicating that the stomatal phenotype observed upon *EXPA1* OE may be regulated by mechanisms independent of hormonal control.

Next, we quantified the number of epidermal cells per square millimeter (**Figure 2C**) and found that the *EXPA1 OE* line had slightly fewer cells than WT and uninduced plants. However, cell size was consistent across the lines (**Figure 2D**). This suggests that the reduced overall size of *EXPA1 OE* plants may result from a lower cell count in the leaves, potentially due to expansins’ role in CW remodeling (see in the text below), which may reduce cell division or limit individual cell expansion.

### *EXPA1* overexpression has strong effect on the *Arabidopsis* shoot morphology

**Figure 3:**
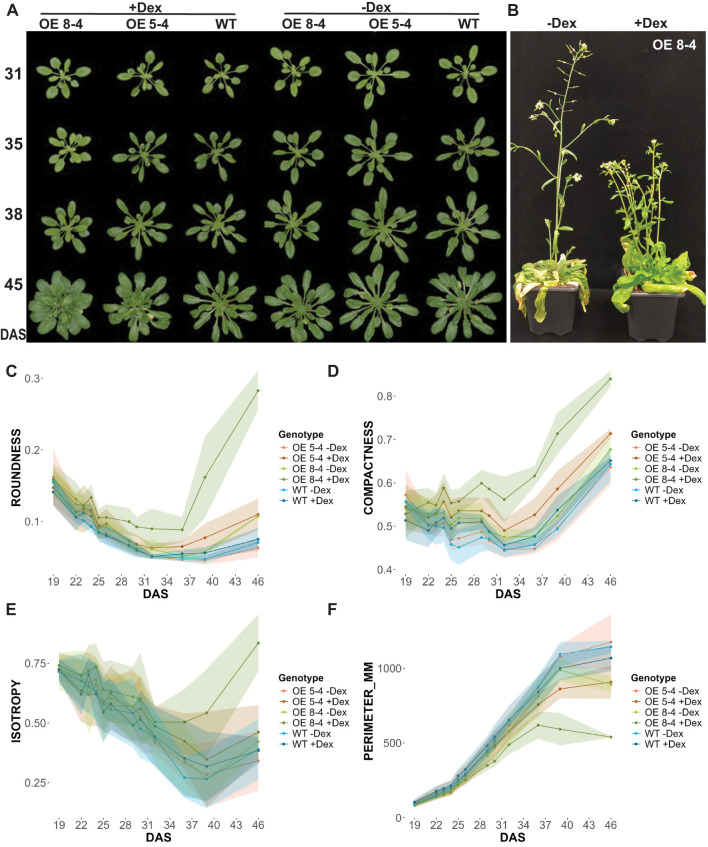
Phenotypic analysis of *EXPA1* overexpressing plants. **(A)** Representative images of wild-type (WT) and *EXPA1 OE* lines 8-4 and 5-4 induced (+Dex) or not (-Dex) with Dex, imaged at specified days after stratification (DAS) and **(B)** at the end of the experiment (app. 10 weeks). Graphs showing parameters from RGB top view imaging **(C)** roundness (arbitrary units, a.u.), **(D)** compactness (a.u.), **(E)** isotropy (a.u.), and **(F)** perimeter (mm) of *EXPA1 OE* (lines 8-4 and 5-4) and WT over time. Highlighted lines represent standard deviation (S.D.) with 16-19 plants per treatment and line analyzed.

To investigate further the phenotypic effects of *EXPA1* OE in shoots, we employed the high-throughput automatic phenotyping platform, Plantscreen™ Compact System (PSI, Photon Systems Instruments), to perform RGB imaging for growth and morphological analysis. Seeds of wild-type (WT) and Dex-inducible *EXPA1 OE* lines (5-4 and 8-4) were sown in pots, with Dex applied 3 times a week. Phenotyping began 10 days after stratification (DAS) and was continued every other day for approximately 10 weeks (**Figures 3A, B**). Distinct morphological changes were observed from the early growth phase, specifically in roundness, compactness and perimeter. These changes became significant at 29 and 32 DAS in the stronger (8-4) and weaker (5-4) *EXPA1 OE* line, respectively (**Figures 3C–F**; **Supplementary Data Sheets S1**, **S2**). Compared to WT, the rosettes of Dex-treated *EXPA1 OE* line 8-4 were rounder, and the rosette leaves had shorter petioles, making the plants appear more compact and symmetric, as reflected by increased isotropy (**Figure 3E**). Uninduced plants, however, resembled WT. By the end of the experiment, the Dex-induced *EXPA OE* line 8-4 exhibited a smaller overall size, more branched inflorescence, delayed senescence, and greener leaves compared to untreated plants (**Figure 3B**). In addition to these changes, Dex-induced 8-4 plants displayed drop in the (pro)cambium activity (thinner procambial layer) associating with reduced inflorescence stem diameter and developed vascular tissue with evident metaxylem, alongside indications of precocious secondary CW formation in the interfascicular regions (**Supplementary Figure S2**).

### *EXPA1* overexpression improves drought tolerance in *Arabidopsis*

**Figure 4:**
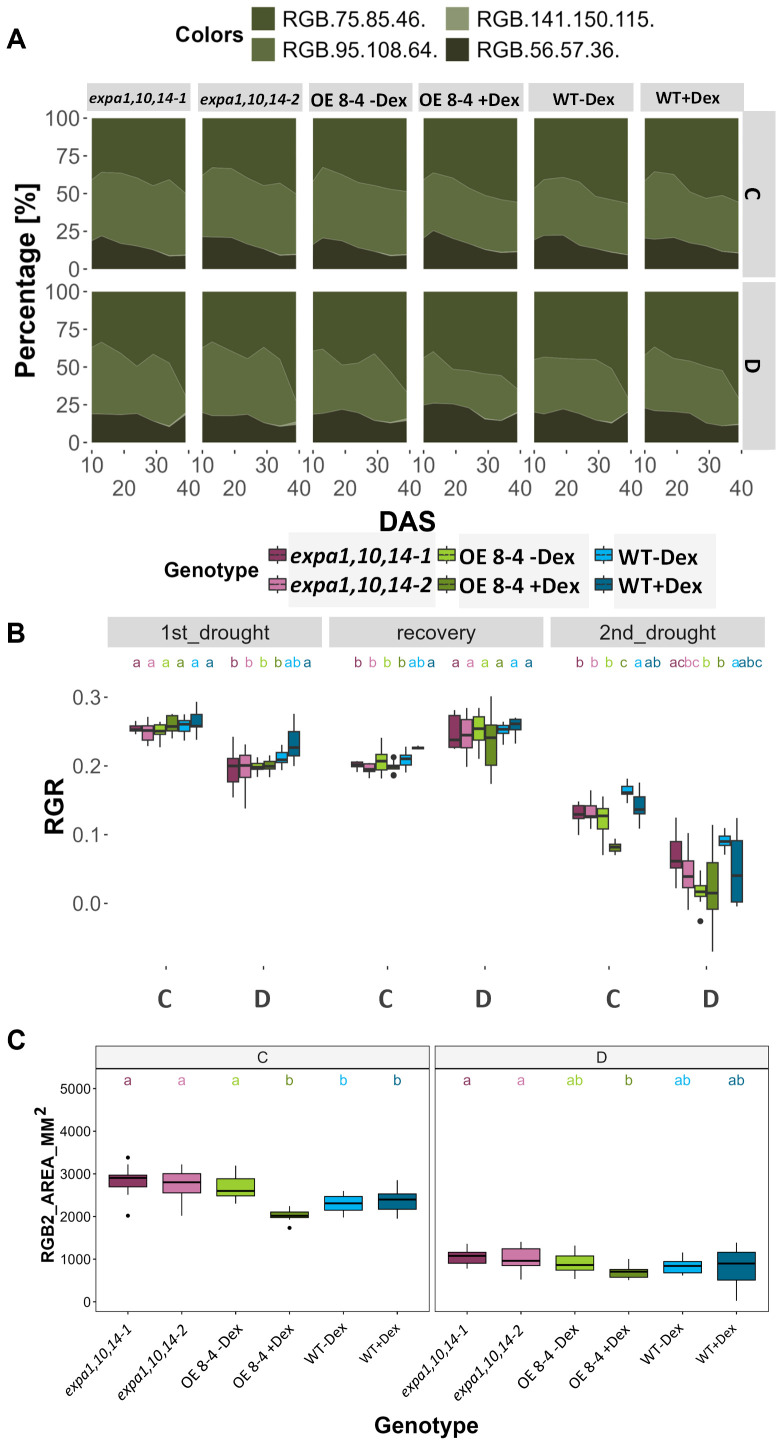
Canopy greenness and growth dynamics under drought stress. **(A)** Plant color segmentation in percentage (%) from canopy top-view images, including four defined hues across all time points (DAS) under control (C) and drought stress (D). **(B)** Relative growth rate (RGR) across three phases: the first drought stress, the second drought stress, and the recovery phase. **(C)** Top- view plant area (mm^2^) at 36 DAS under control and drought stress. Significant differences among lines per DAS were assessed using the Kruskal-Wallis test, with differing lowercase letters above the boxplot indicating statistical significance (p < 0.05).

To evaluate the morphological and physiological response of soil-grown plants under drought conditions, triple mutants (lines *expa1,10,14-1* and *2*), together with *EXPA1 OE* line (8-4) and WT plants treated with or without Dex, were phenotyped. Using RGB top-view imaging, the greenness of plant canopy, and growth dynamics, including relative growth rate (RGR) and area, were assessed over time (**Figure 4**; **Supplementary Figure S3**, **Supplementary Data Sheets S1**, **S2**). Across all lines, drought stress (D) reduced both the area and relative growth rate, with an increase in dark green color, the latter becoming particularly severe during the second drought phase compared to control conditions (C). The percentage of light green segmentation (RGB (95,108,64)) and RGR decreased dramatically after the second drought stress phase, reflecting chlorophyll degradation and a consequent reduction in growth (**Figures 4A, B**). Notably, the Dex-induced *EXPA OE* line 8-4 exhibited a smaller reduction in the percentage of darker green segments, suggesting reduced stress-induced tissue degradation. This line also showed slower RGR over time and a consistently smaller area during the late stress phase, including 36 DAS, regardless of drought treatment (**Figure 4C**). Importantly, growth in WT was not affected by Dex treatment, as both treated and untreated plants were of similar size. Interestingly, the results indicated that the triple mutant lines had significantly larger areas compared to WT (**Figure 4C**; **Supplementary Figure S3**).

**Figure 5:**
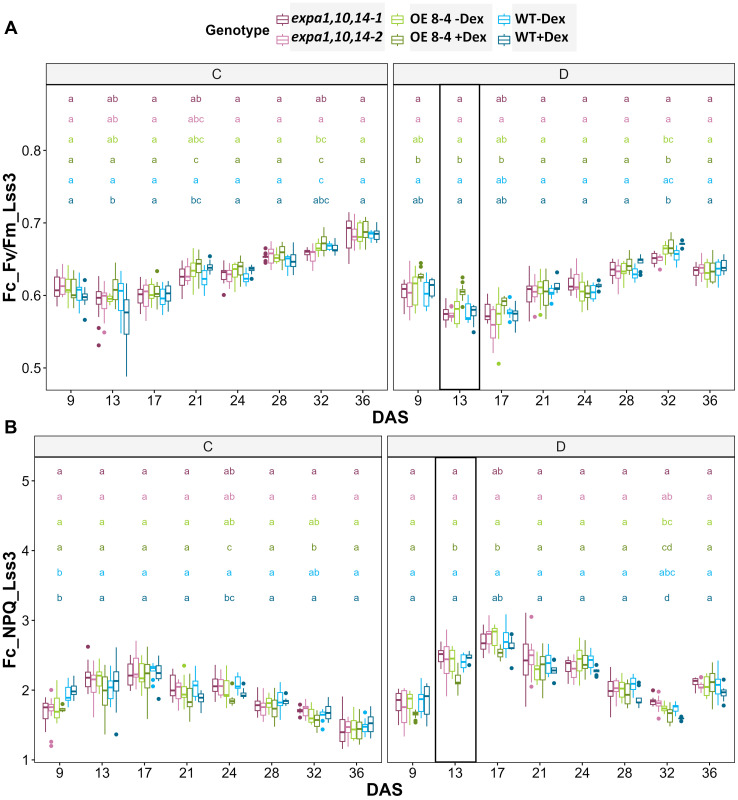
Photosynthetic efficiency under drought stress. **(A)** PSII maximum efficiency of light-adapted plants in light steady-state (F_V_/F_M__Lss) and **(B)** non-photochemical quenching steady-state (NPQ_Lss) at 320 µmol m^−2^ s^−1^ (Lss3) which is the third light level measured using light response curve protocol across multiple time points. Significant differences among lines at each DAS were assessed using the Kruskal-Wallis test, with differing lowercase letters above the boxplot indicating statistical significance (p < 0.05).

Furthermore, chlorophyll fluorescence (ChlF) measurements were conducted using an optimized light-response curve protocol with four light steady-state levels (Lss1-Lss4) (**Supplementary Figure S4**). Key ChlF parameters measured included minimal fluorescence (F_0_) and maximal fluorescence (F_M_), from which variable fluorescence (F_V_) was calculated. Additionally, photochemical (qP_Lss) and non-photochemical quenching (NPQ_Lss) in light steady state were assessed (**Supplementary Table S1**). These measurements provide important insights into the functionality of Photosystem II (PSII) and allow to evaluate photosynthetic efficiency. Significant differences in photosynthetic efficiency under stress conditions were observed at light steady state 3 and 4 (Lss3 and Lss4) of the light-response curve (**Supplementary Data Sheet S2**). At the early phase of drought stress (13 DAS), the Dex-induced *EXPA1 OE* line exhibited higher F_V_/F_M_ and lower NPQ value at Lss3 (**Figure 5**). This increased photosynthetic efficiency, indicating enhanced performance under drought conditions, suggests that *EXPA1* OE improves stress tolerance by modulating PSII efficiency and photoprotective mechanisms. These findings highlight the potential of *EXPA1* as a target for improving drought tolerance in plants.

**Figure 6:**
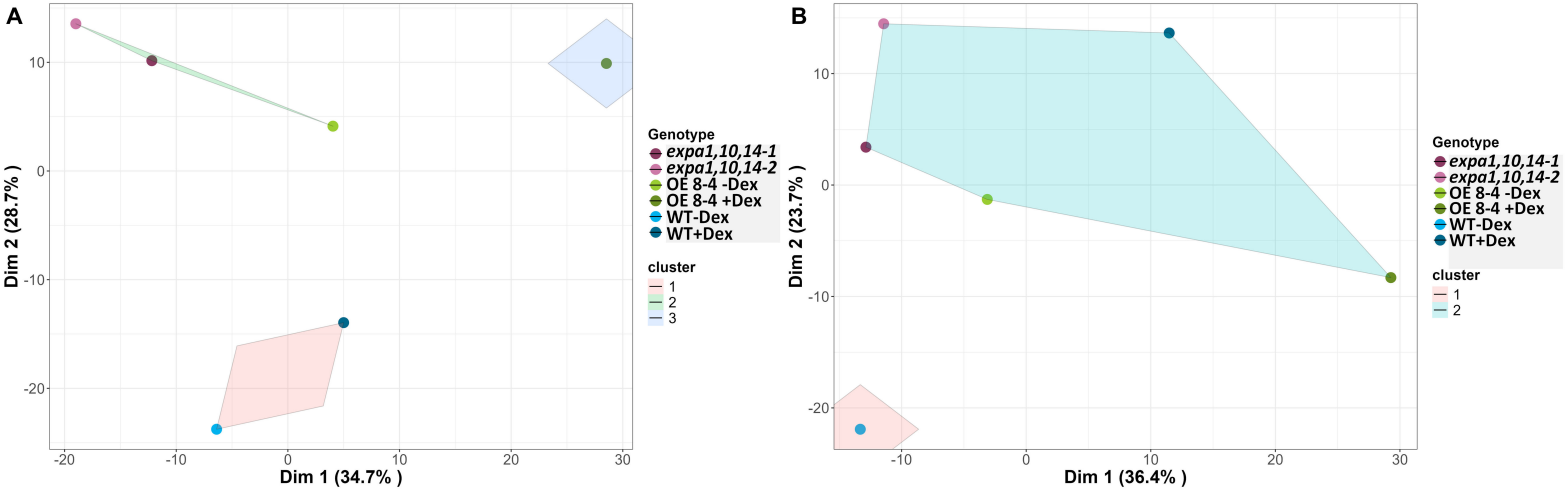
Visualization of k-means clustering presented on a two-dimensional PCA plot. **(A)** Control and **(B)** drought conditions. Lines are color-coded, and shaded convex hulls represent the grouping of lines based on k-means clustering.

For integrated data visualization, Random Forest models were applied with a mean accuracy of 0.83-0.89 across all time points to investigate the contribution of various RGB-based and fluorescence-related traits in distinguishing between genotypes under control and drought conditions, respectively (**Supplementary Figures S5A, B**). A total of 34 traits were measured and used in the Random Forest analysis, with the top 20 traits selected for further investigation. Morphological traits were found to be more important during the early phase, while physiological traits gained higher significance in the later phase, reflecting genotype-specific responses at different time points. Furthermore, analysis of the 20 selected traits (variables) for3discriminating between treatments across all genotypes (**Supplementary Figure S5C**) revealed that area and compactness contributed the most to treatment prediction, followed by NPQ_Lss4 and QY_max. Additionally, k-means clustering was visualized on a two-dimensional Principal Component Analysis (PCA) plot for all measured parameters across all time points to investigate the overall performance of the lines (**Figure 6**). The results showed the *EXPA1 OE* line (8-4) exhibited different mechanisms when induced with dex, particularly under control conditions (**Figure 6A**). The WT clustered together under control conditions but diverged under drought stress (**Figure 6B**). In contrast, the triple mutant lines (*expa1,10,14-1 and -2)* consistently clustered together regardless of the treatment.

### *EXPA1* overexpression induces changes in cell wall-related gene expression

To investigate the molecular mechanisms underlying *EXPA1*-induced phenotypic changes, we performed a genome-wide transcriptomic analysis to monitor changes at both early (3 hours) and late (7 days) stages following *EXPA1* OE in *Arabidopsis* seedling shoots. Differentially expressed genes (DEGs) were identified based on an adjusted p-value of < 0.05 and a log2FoldChange threshold of ±1. At 3 h post-Dex induction of *EXPA1* OE, 995 genes were significantly upregulated and 1114 genes were downregulated in the *EXPA1 OE* line compared to non-induced controls (Dex-treated WT, **Supplementary Data Sheet S3**). Among the DEGs, several plant-type CW-related Gene Ontology (GO) terms were significantly enriched. These included (plant-type) CW loosening (GO0009828), CW organization (GO0009664), or CW biogenesis (GO0071669). In addition to the intentionally overexpressed *EXPA1* gene, several other expansin and expansin-like (*EXL*) genes showed altered expression (**Supplementary Table S2**). Notably, the expression of *EXPA2*, *EXPA7* and *EXLA2* was upregulated, while *EXPA6*, *EXPA8*, *EXPA15*, *EXPB3* and *EXLB1* were downregulated (**Table 2**). Other CW-related genes, including *XYLOGLUCAN: XYLOGLUCOSYL TRANSFERASES* (*XTH*), *PECTIN METHYLESTERASES* (*PME*), and *CELLULOSE SYNTHASE-LIKE* (*CSL*) genes, were also significantly enriched among the DEGs (**Table 2**). At 7 days post-Dex induction of *EXPA1* OE, 637 genes were up regulated and 522 were downregulated compared to the Dex-treated WT control (**Supplementary Data Sheet S3**). In contrast to the short-time response, only the GO terms related to plant-type CW loosening remained significantly enriched, and fewer CW-related genes were differentially expressed (**Table 3**). Dex treatment alone did not cause significant changes in plant growth and development, and only 11 genes were upregulated and 14 were downregulated.

**Table 2:** CW-related DEGs identified 3 h after Dex-induced *EXPA1* overexpression.

8-4 Dex3h vs WT Dex3h		baseMean	log2FC	stat	padj
EXPA1	AT1G69530	449557.3	5.6	77.9	0.000
EXPA2	AT5G05290	95.2	2.8	11.6	0.000
EXPA6	AT2G28950	2793.7	-1.2	-12.0	0.000
EXPA7	AT1G12560	68.5	1.2	4.0	0.000
EXPA15	AT2G03090	524.4	-1.4	-8.5	0.000
EXPB3	AT4G28250	560.2	-1.9	-12.2	0.000
EXLA2	AT4G38400	763.5	1.8	11.8	0.000
EXLB1	AT4G17030	103.3	-1.7	-8.6	0.000
XTH12	AT5G57530	43.7	1.3	2.8	0.014
XTH15	AT4G14130	5054.7	4.0	59.9	0.000
XTH16	AT3G23730	3377.1	1.2	22.7	0.000
XTH17	AT1G65310	860.8	1.4	5.7	0.000
XTH18	AT4G30280	2356.5	2.3	7.6	0.000
XTH19	AT4G30290	1998.7	1.7	13.1	0.000
XTH22	AT5G57560	7680.2	1.2	4.9	0.000
XTH23	AT4G25810	1255.0	2.1	9.5	0.000
XTH25	AT5G57550	46.6	-1.5	-5.3	0.000
XTH31	AT3G44990	1148.9	-1.3	-9.6	0.000
PME3	AT3G14310	8506.1	-1.1	-11.1	0.000
PME20	AT2G47550	137.1	1.7	6.8	0.000
PME24	AT3G10710	54.7	1.0	3.2	0.004
PME46	AT5G04960	112.1	1.7	5.9	0.000
PME53	AT5G19730	345.0	-1.1	-7.9	0.000
CSLA11	AT5G16190	192.3	1.2	7.4	0.000
CSLB3	AT2G32530	737.4	-1.6	-15.6	0.000
CSLB4	AT2G32540	1396.8	-1.2	-14.8	0.000
CSLB06	AT4G15320	52.9	-1.7	-5.8	0.000
CSLC12	AT4G07960	299.5	1.6	13.7	0.000
CSLD5	AT1G02730	3698.9	-1.1	-18.4	0.000
CSLE1	AT1G55850	6112.9	-1.7	-9.0	0.000
CSLG3	AT4G23990	203.0	-2.1	-14.8	0.000

**Table 3:** CW-related DEGs identified 7 d after Dex-induced *EXPA1* overexpression.

8-4 Dex7d vs WT Dex7d		baseMean	log2FC	stat	padj
EXPA1	AT1G69530	331384.7	5.1	25.6	0.000
EXPA2	AT5G05290	97.2	3.3	8.3	0.000
EXPA8	AT2G40610	1753.7	-1.4	-15.3	0.000
EXPA15	AT2G03090	1131.8	-1.5	-5.3	0.000
EXPB1	AT2G20750	150.3	-1.5	-3.1	0.014
EXPB3	AT4G28250	3151.8	-1.1	-3.1	0.014
EXLA1	AT3G45970	532.4	1.2	3.4	0.006
EXLB1	AT4G17030	335.5	-1.4	-4.1	0.001
XTH10	AT2G14620	87.7	1.3	7.2	0.000
XTH15	AT4G14130	494.3	1.4	3.9	0.001
XTH17	AT1G65310	278.4	-1.3	-5.6	0.000
XTH23	AT4G25810	611.0	1.4	3.0	0.018
XTH24	AT4G30270	3767.8	1.5	6.4	0.000
PME13	AT2G26450	52.7	5.2	10.6	0.000
PME18	AT1G11580	3481.5	1.2	3.4	0.006
PME61	AT5G53370	4636.7	1.0	3.9	0.001

Altogether, our findings indicate that *EXPA1* overexpression triggers rapid and extensive changes in the expression of numerous CW-related genes, particularly during the early stages of induction.

## Discussion

### Expansin expression in shoot epidermis and specialized cells

Expansins are well-documented for their role in CW loosening, facilitating cell expansion and growth of specific cells, tissues and organs (Cosgrove, 2000a; c). Several bioinformatics resources dedicated to the expansin gene family provide valuable tools for the navigation and classification of expansins and their homologues (Lohoff et al., 2020; Kok et al., 2023). Beyond CW modification, expansins are involved in various biological processes, reflecting the diversity in protein sequences and gene expression patterns across developmental and stress-related contexts (reviewed in Cosgrove, 2024; Marowa et al., 2016; Samalova et al., 2022).

Recently, cell-specific localization of expansins was demonstrated using red fluorescent protein fusions in roots (Samalova et al., 2024). Building on this approach, our study reveals predominant expression of *EXPA1* in stomatal guard cells, suggesting a specific function in these specialized cells that respond dynamically to environmental stress. Earlier studies anticipated this expression pattern by using promoter-driven reporter gene (Cosgrove, 2000c) and demonstrated that the guard cell expansin *AtEXPA1* regulates stomatal opening (Zhang et al., 2011). Additional expansins examined in this study, including EXPA10, EXPA14, and EXPA15, were predominantly localized to the CWs of the shoot epidermis, suggesting a role in modulating shoot structure and function. Notably*, EXPA15* displayed strong expression throughout the leaf epidermis and in various specialized cells, such as those at the trichome base, the shoot apical meristem, and petal leaves. Our attempts to generate an *EXPA15* mutant using CRISPR/Cas9 were unsuccessful, possibly highlighting its essential role in plant growth and development.

### Changes in CW dynamics due to *EXPA1* overexpression

Our transcriptomic analysis provides insights into the downstream effects of *EXPA1* OE on CW dynamics. Early induction of *EXPA1* altered the expression of numerous CW-related genes, including those encoding XTH, PME, and CSL proteins, which play key roles in CW restructuring. Moreover, several other expansin genes were either upregulated or downregulated. These rapid shifts in gene expression suggest that *EXPA1* may act as a regulatory node within CW-associated gene networks, impacting CW composition, structure, and function. This is in line with our previous findings, suggesting changes in the transcription of CW-modifying genes as one of the possible mechanisms leading to EXPA1-induced CW stiffening in the root (Samalova et al., 2024). Notably, recent transcriptomic studies identified *EXPA1* as a primary regulator under stress conditions, supporting this regulatory role (Sanchez-Munoz et al., 2024). However, similarly to the situation in the root, also in the shoot we observed specific localization of individual EXPAs, implying position-specific mode of action. Thus, we cannot exclude that the transcriptome changes observed in the *EXPA1 OE* lines might be (at least partially) indirect, balancing the ectopic localization of EXPA1.

The coordinated action of expansins thus appears essential not only for growth under standard conditions but also for adaptation to stressful environments, as we observed in the context of drought. This aligns with previous studies indicating that expansins act synergistically with other CW modifiers to create a more dynamic, responsive CW environment (Cosgrove, 2005). Expansins not only act as CW-loosening agents but may also orchestrate CW properties and composition by interfering with the action of CW remodeling enzymes, possibly through both carbohydrate-dependent and -independent mechanisms (Samalova et al., 2022). This dual role enables plants to maintain CW homeostasis, balancing wall flexibility needed for growth with structural integrity during stress.

In addition to modifying CW biomechanics, composition and structure, cell wall integrity (CWI) likely plays a crucial role, potentially triggering wall integrity signaling through receptor kinases that restrict growth following wall disruptions (Gigli-Bisceglia et al., 2020; Wolf, 2022). CWI sensing initiates stress responses, including ROS production, hormone signaling (e.g., jasmonic acid, salicylic, ethylene), and structural reinforcements (Hamann, 2015; Novakovic et al., 2018). Feedback regulation of CWI may modulate *EXPA1*-induced wall remodeling by activating protective responses during instances of excessive wall strain. However, the exact mechanisms through which CWI signaling interfaces with hormonal or stress pathways remain to be fully elucidated (Vaahtera et al., 2019; Rui and Dinneny, 2020).

### Phenotypic changes linked to *EXPA1* overexpression

We observed that *EXPA1* OE not only alters gene expression related to CW remodeling but also leads to distinct phenotypic changes in shoot architecture, potentially enhancing stress tolerance. Phenotypic assessments revealed that *EXPA1* OE contributes to notable morphological alterations, including modified rosette architecture, an increased number of inflorescence stems with reduced diameter, and apparent delayed senescence, all of which may influence the plant’s ability to cope with environmental stress. These modifications suggest that EXPA1 impacts both structural and developmental processes in the shoot. Specifically, the compact rosette morphology and a highly branched inflorescence in *EXPA1* OE plants may indicate a shift in resource allocation, potentially enhancing reproductive growth under stress conditions. Our findings align with those of Dai et al. (2012), who observed that expansin OE promotes growth and reproductive development, possibly by enhancing resource use efficiency.

However, our observation that *EXPA1* OE plants are smaller, likely due to a reduced number of epidermal cells in the leaves, contradicts previous studies. Kwon et al. (2008) reported that OE of *AtEXP3* increases leaf size and growth under normal conditions. Similarly, Kuluev et al. (2012) demonstrated that *AtEXPA10* OE leads to larger cells and leaves, underscoring expansins’ role in cell enlargement and tissue architecture. Additionally, Chen et al. (2018) showed that RNAi plants exhibit smaller leaf size, with epidermal cells that appear to be smaller, more compact with a higher density per mm^2^ leaf area compared to *EXPA4* OE tobacco lines and WT. In our study, however, the epidermal cells in *EXPA1 OE* plants were not smaller or denser. Instead, it appears that the smaller phenotype results from a reduced number of cells in leaves, a pattern we previously observed in roots (Samalova et al., 2024), potentially due to expansins’ role in CW remodeling, which may limit cell division. This is strongly evidenced by the reduced procambium and impaired radial expansion observed in *EXPA1* OE inflorescence stems. Further supporting our findings, we observed that the triple knockout of *expa1expa10expa14* generated in this study displayed an unexpectedly larger phenotype based on phenotypic RGB analysis of the rosette.

One of the most intriguing aspects of *EXPA1* OE described in this study is the increase in stomatal density and aperture size, providing new insights into the role of expansins in modulating stomatal behavior. The stomata in Dex-induced *EXPA1 OE* plants appear fully functional, responding effectively to ABA-induced closure. These alterations likely enhance the plant’s ability to respond to environmental signals by enabling more dynamic stomatal responses to environmental cues, along with improved water retention capacity under fluctuating conditions. This model aligns with findings by Wei et al. (2011) and Zhang et al. (2011), who demonstrated that OE of *AtEXPA1* and *VfEXPA1* accelerates light-induced stomatal opening suggesting that expansins modulate stomatal movement by influencing guard CW extensibility. This is consistent with the reduced drought tolerance observed in expansin-inhibited lines, where stomatal responsiveness to environmental stimuli was also reduced, leading to lower gas exchange efficiency (Zhang et al., 2011; Lu et al., 2013).

To sum up, ours as well as others data suggest that *EXPA1* OE not only impacts stomatal traits but also regulates cell division and expansion, resulting in altered cellular and physiological characteristics in *Arabidopsis*. Further research is needed to elucidate the molecular pathways involved in these processes.

### Drought tolerance via modulation of guard cells and CW composition

A recent review of 22 studies found that OE of expansins across various plant species enhances tolerance to abiotic stresses such as drought, salt, cold, heat, light, cadmium, and oxidative stress (Samalova et al., 2022). Although the mechanisms underlying expansin-induced stress tolerance are not fully understood, they likely involve CWI signaling (Gigli-Bisceglia et al., 2020), ROS signaling (Tenhaken, 2015), improved cell integrity (Chen et al., 2018), and increased wall flexibility under water-deficit conditions, all of which may alter CW and membrane properties (Narayan et al., 2019; Lu et al., 2013). Our study indicates that *EXPA1* OE impacts the overall transcriptional states, which may influence not only CW metabolism and biomechanics (Samalova et al., 2024; Cosgrove, 2024), but also modifies plant structure and stomatal function enhancing the ability of shoots to respond and withstand low-water conditions.

Our results demonstrate that *EXPA1* OE in *Arabidopsis* contributes to drought tolerance, as evidenced by chlorophyll fluorescence measurements, including the increased maximum efficiency of PSII, which quantifies the conversion of light energy into chemical energy during photosynthesis (F_V_/F_M_) and NPQ values, which measure how excess light energy is dissipated as heat to protect plants from photoinhibition. Lower NPQ values observed in the *EXPA1* OE lines under drought conditions, which indicate increased stress tolerance, suggest that *EXPA1* OE plants are in a better physiological state that aids in maintaining photoprotection while reducing energy dissipation as heat, allowing for greater energy conservation during recovery processes. Overexpression lines also showed enhanced chlorophyll fluorescence recovery following drought stress, specifically through improved PSII efficiency, which is critical for maintaining photosynthetic performance under water deficit conditions. Similar enhancements in photosynthesis have been observed in other expansin OE lines, such as *AtEXPA1* and *VfEXPA1*, which led to increased transpiration and photosynthesis rates under favorable light conditions (Wei et al., 2011; Zhang et al., 2011).

Previously, an association between stomata density and morphology and water use efficiency was demonstrated (Bertolino et al., 2019). Reduced stomatal density often enhances water-use efficiency, and engineering crops with lower stomatal densities was shown to improve drought tolerance without yield penalties, however, these responses appear to be species-specific and context-dependent (Bertolino et al., 2019; Hepworth et al., 2015). Thus, the higher stomata density and diameter observed in *EXPA1 OE* plants may contribute to the changes in drought adaptation under drought conditions. This response may be facilitated by changes in CW rigidity and hydraulic through expansin activity, promoting water retention, as seen in tobacco OE *NtEXPA11* (Marowa et al., 2020) and optimizing gas exchange in a stress-resilient manner (Farooq et al., 2009).

Together, these findings emphasize the functional role of expansins in optimizing the balance between water conservation and photosynthetic efficiency under drought stress.

### Expansin-based strategies for enhancing crop resilience and productivity

The results of this study have important implications for addressing global agricultural challenges, particularly in the context of climate change and the growing demand for sustainable food production. As climate change intensifies droughts and exacerbates water scarcity, the development of climate-resilient crops has become increasingly critical. The demonstrated ability of *EXPA1* OE to improve drought tolerance in *Arabidopsis* suggests that manipulating expansin expression represents a promising strategy for improving water-use efficiency in crops. Furthermore, the role of EXPA1 in CW remodeling and plant growth highlights its potential for enhancing biomass production and crop yield. With the global population on the rise, boosting crop productivity without requiring additional land or resources is essential to ensure better harvests and minimize crop losses during extreme weather events such as storms and strong winds. Due to the unique localization of EXPA1 in stomatal guard cells, this study suggests that *EXPA1* manipulation could also improve crop resilience against biotic stresses, especially pathogens such as bacteria and fungi, that enter via these structures. Such an approach could reduce the need for chemical inputs, promoting more sustainable agricultural practices.

Based on our findings and recent literature, we propose that modifying *EXPA1* expression, or other α-expansins (*EXPA*), can confer multiple desirable traits in crops, significantly enhancing agricultural productivity, biomass production, stress tolerance, and disease resistance. Especially fine-tuning *EXPA* expression by controlling its timing and tissue specificity holds significant potential for improving various crop traits. Given that *EXPA1* is involved in CW loosening and remodeling, modulating its expression could promote plant growth and overall biomass accumulation. This could be particularly advantageous in crops where increased growth directly correlates with enhanced yield potential. For example, influencing grain size and quality traits, as seen with *OsEXPA7* in rice (*Oryza sativa*) (Zhang et al., 2024) and *TaExpA6* in wheat (*Triticum aestivum*) (Calderini et al., 2021), or growth, lignification and fiber cell elongation, as demonstrated by OE of *PagEXPA1* in poplar (*Populus alba x Populus glandulosa*) (Hao et al., 2024). EXPA modulation can also improve biomass digestibility and industrial processing (Mira et al., 2024) and improve fiber quality as shown for EXPA1 in cotton (Yaqoob et al., 2020). In crops such as wheat or oilseed crops, adjusting the expression of *EXPA* could influence stem rigidity and height, which is important for preventing lodging. In oil palm breeding programs, for instance, shortening the stem has been beneficial for producing semi-dwarf palms that are easier to harvest (Somyong et al., 2022). Modifying *EXPA* expression in fruits could impact ripening and texture, which are critical factors for post-harvest storage. For instance, the simultaneous knockout of *SlEXP1* and *SlCEL2* in tomato (*Solanum lycopersicum*) enhanced fruit firmness and increased cell adhesion (Su et al., 2024).

Overexpression of *EXPA* in roots could enhance root growth and nutrient uptake, particularly valuable in nutrient-poor or stressed soils. For example, OE of *GmEXPA7* in soybean (*Glycine max*) hairy roots enhanced low phosphorus tolerance by stimulating root growth and improving phosphorus uptake (Liu et al., 2024). Additionally, *EXPA* OE has been shown to improve tolerance to heavy metals in various species, such as the enhancement of aluminum tolerance in composite carpet grass (*Axonopus compressus*) through the manipulation of *AcEXPA1* (Li et al., 2024) or cadmium in tobacco through *TaEXPA2* OE (Ren et al., 2018). This trait could be applied to crops grown in contaminated soils, offering potential for phytoremediation. Finally, enhancing *EXPA* expression in roots could also improve resistance to soil-borne pathogens, such as root-knot nematodes, as observed with *NtEXPA7* OE in tobacco (Yang et al., 2024).

In conclusion, modifying *EXPA* expression, not only in terms of levels but also in terms of spatiotemporal dynamics, offers a versatile tool for improving multiple crop traits, ranging from drought resistance and pathogen defense to biomass production, grain yield, and stress tolerance under challenging growing conditions. Integrating *EXPA1* into crop breeding and biotechnological applications could play a pivotal role in developing resilient, high-yielding varieties suited to the challenges of climate change. This approach provides an opportunity to empower farmers with robust cultivars capable of thriving in increasingly unpredictable environments.

## Conclusions

Our study highlights the multifaceted role of EXPA1 in influencing growth, modulating CW properties, and enhancing drought tolerance in *Arabidopsis*, offering insights into how expansins facilitate adaptation to environmental stress through guard cell dynamics, CW restructuring, and improved photosynthetic efficiency under water-limited conditions. The data suggest that expansins play a central role in the plant’s drought response by coordinating growth and development through guard cell modulation and CW remodeling. Additionally, the diversity in expansin gene expression across different plant cells further underscores their varied roles throughout developmental stages and in response to environmental conditions.

This research adds to the evidence supporting expansins’ role in drought tolerance and presents a pathway for engineering crops with enhanced resilience to abiotic stress. The potential of EXPA1-mediated CW modifications and guard cell dynamics opens a path toward improving drought tolerance in crops through targeted manipulation of specific expansin genes, contributing to sustainable agriculture in changing climates.

Future research could further explore the molecular interactions of EXPA1 with other CW-modifying enzymes and investigate expansin gene manipulation across various crops to optimize yield, drought tolerance, and stress resilience.

## Data Availability

The data presented in the study are deposited in the NCBI’s Gene Expression Omnibus repository, accession number GSE286232.

## Supplementary material

Supplementary Figure 1 Dose-response effect of osmotic stress on *Arabidopsis* seedlings grown *in vitro.* Graphs represent **(A)** germination rate (%), **(B)** root length (mm), and **(C)** fresh weight (mg) of shoots of WT, *expansin* mutants (*expa1-2*, *expa10-1* and *-2*, *expa1,10-1* and *-2*, *expa10,14-1* and *-2*, *expa1,10,14-1* and *-2*) and *EXPA1 OE* line (8-4; -Dex and +Dex) grown on MS media supplemented with various concentrations of NaCl (as indicated) for 7 days. Error bars represent S.D., with at least 10 plants per treatment and line analyzed.

Supplementary Figure 2
*EXPA1* overexpression reduces the diameter of *Arabidopsis* stem. Plants of wild-type (WT) and Dex-inducible *EXPA1 OE* line 8-4 were grown in soil as described for the control condition experiment and either treated with Dex (+Dex) or left untreated (-Dex) until app. 10-week-old. Inflorescence stem cross-sections were prepared using a vibratome and analyzed as follows: **(A)** sections stained with toluidine blue visualized using Olympus BX61 microscope, with a detailed view in **(B)**. **(C)** Sections stained with calcofluor white in green (excitation 458 nm/detection 499-579 nm) imaged alongside autofluorescence in red (excitation 561 nm/detection 588-678 nm) using a Zeiss 880 CLSM. The arrows point at possible onset of precocious secondary CW formation in the interfascicular regions. Scale bars correspond 0.5 mm in **(A)** and 50 μm in **(B, C)**.

Supplementary Figure 3 Plant area from RGB top view-imaging along DAS. Solid lines represent stress induction phases, where D1 and D2 correspond to the first and second drought stress phases, respectively, and R indicates the recovery phase. The data represent mean values, with shaded areas indicating standard deviation (S.D.).

Supplementary Figure 4 Light response curve using kinetic chlorophyll fluorescence imaging. **(A)** PSII maximum efficiency of light-adapted sample (F_V_/F_M__Lss), **(B)** PSII quantum yield at light steady-state indicating operating efficiency of PSII (QY_Lss), **(C)** Non-photochemical quenching at steady-state (NPQ_Lss), **(D)** Coefficient of non-photochemical quenching in light steady state (qN_Lss), **(E)** Coefficient of photochemical quenching in steady-state (qP_Lss) and **(F)** Fraction of PSII centers that are ‘open’ in steady state (qL_Lss). Data were collected under four light levels at 13 DAS.

Supplementary Figure 5 Variable importance of traits identified by the Random Forest model across all time points. Morphological and physiological traits that contributed the most to discriminating between the genotypes under **(A)** control and **(B)** drought conditions at specific DAS. **(C)** Key traits of high importance that differentiate between treatments among all lines. The mean accuracy metric indicates the prediction accuracy of the random forest model.

Supplementary Table 1 Trait description extracted from the RGB and chlorophyll fluorescence imaging.

Supplementary Table 2 GO terms significantly enriched among DEGs identified 3 h after Dex-induced *EXPA1* overexpression.

Supplementary Data Sheet 1 Stats all letters.

Supplementary Data Sheet 2 Stats non parameter pvalues.

Supplementary Data Sheet 3 RNAseq shoots.

## References

[B1] Awlia M. Nigro A. Fajkus J. Schmoeckel S. M. Negrao S. Santelia D.. (2016). High-throughput non-destructive phenotyping of traits that contribute to salinity tolerance in *Arabidopsis thaliana* . Front. Plant Sci. 7. doi: 10.3389/fpls.2016.01414 PMC5039194 27733855

[B2] Bertolino L. T. Caine R. S. Gray J. E. (2019). Impact of stomatal density and morphology on water-use efficiency in a changing world. Front. Plant Sci. 10. doi: 10.3389/fpls.2019.00225 PMC6414756 30894867

[B3] Calderini D. F. Castillo F. M. Arenas A. Molero G. Reynolds M. P. Craze M.. (2021). Overcoming the trade-off between grain weight and number in wheat by the ectopic expression of expansin in developing seeds leads to increased yield potential. New Phytol. 230, 629–640. doi: 10.1111/nph.17048 33124693 PMC8048851

[B4] Chaves M. M. Flexas J. Pinheiro C. (2009). Photosynthesis under drought and salt stress: regulation mechanisms from whole plant to cell. Ann. Bot. 103, 551–560. doi: 10.1093/aob/mcn125 18662937 PMC2707345

[B5] Chen Y. Han Y. Zhang M. Zhou S. Kong X. Wang W. (2016). Overexpression of the wheat expansin gene *TaEXPA2* improved seed production and drought tolerance in transgenic tobacco plants. PloS One 11, e0153494. doi: 10.1371/journal.pone.0153494 27073898 PMC4830583

[B6] Chen L. Zou W. Fei C. Wu G. Li X. Lin H.. (2018). [amp]]alpha;-Expansin *EXPA4* positively regulates abiotic stress tolerance but negatively regulates pathogen resistance in *Nicotiana tabacum* . Plant Cell Physiol. 59, 2317–2330. doi: 10.1093/pcp/pcy155 30124953

[B7] Cho H. T. Cosgrove D. J. (2000). Altered expression of expansin modulates leaf growth and pedicel abscission in Arabidopsis thaliana. Proc. Natl. Acad. Sci. 97, 9783–9788. doi: 10.1073/pnas.160276997 10931949 PMC16942

[B8] Cho H. T. Cosgrove D. J. (2002). Regulation of root hair initiation and *EXPANSIN* gene expression in *A. thaliana* . Plant Cell 14, 3237–3253. doi: 10.1105/tpc.006437 12468740 PMC151215

[B9] Clauw P. Coppens. F. De Beuf K. Dhondt S. Van Daele T. Maleux K.. (2015). Leaf responses to mild drought stress in natural variants of *Arabidopsis* . Plant Physiol. 167, 800–816. doi: 10.1104/pp.114.254284 25604532 PMC4348775

[B10] Clough S. J. Bent A. F. (1998). Floral dip: a simplified method for *Agrobacterium* mediated transformation of *Arabidopsis thaliana* . Plant J. 16, 735–743. doi: 10.1046/j.1365-313x 10069079

[B11] Cosgrove D. J. (2000a). Loosening of plant cell walls by *EXPANSIN*s. Nature 407, 321–326. doi: 10.1038/35030000 11014181

[B12] Cosgrove D. J. (2000b). Expansive growth of plant cell walls. Plant Physiol and. Biochem 38, 109–124. doi: 10.1016/S0981-9428(00)00164-9 11543185

[B13] Cosgrove D. J. (2000c). New genes and new biological roles for expansins. Curr. Opin. Plant Biol. 3, 73–78. doi: 10.1016/S1369-5266(99)00039-4 10679451

[B14] Cosgrove D. J. (2005). Growth of the plant cell wall. Nat. Rev. Mol. Cell Biol. 6, 850–861. doi: 10.1038/nrm1746 16261190

[B15] Cosgrove D. J. (2015). Plant *EXPANSIN*s: diversity and interactions with plant cell walls. Curr. Opin. Plant Biol. 25, 162–172. doi: 10.1016/j.pbi.2015.05.014 26057089 PMC4532548

[B16] Cosgrove D. J. (2016). Plant cell wall extensibility: connecting plant cell growth with cell wall structure, mechanics, and the action of wall-modifying enzymes. J. Exp. Bot. 67, 463–476. doi: 10.1093/jxb/erv511 26608646

[B17] Cosgrove D. J. (2024). Plant cell wall loosening by expansins. Annu. Rev. Cell Dev. Biol. 40, 329–352. doi: 10.1146/annurev-cellbio-111822-115334 38724021

[B18] Cosgrove D. J. Li L. C. Cho H. T. Hoffmann-Benning S. Moore R. C. Blecker D. (2002). The growing world of expansins. Plant Cell Physiol. 43, 1436–1444. doi: 10.1093/pcp/pcf180 12514240

[B19] Cutler S. R. Rodriguez P. L. Finkelstein R. R. Abrams S. R. (2010). Abscisic acid: emergence of a core signaling network. Ann. Rev. Plant Biol. 61, 651–679. doi: 10.1146/annurev-arplant-042809-112122 20192755

[B20] Dai F. Zhang C. Jiang X. Kang M. Yin X. Lu P.. (2012). *RhNAC2* and *RhEXPA4* are involved in the regulation of dehydration tolerance during the expansion of rose petals. Plant Physiol. 160, 2064–2082. doi: 10.1104/pp.112.207720 23093360 PMC3510132

[B21] Eden E. Navon R. Steinfeld I. Lipson D. Yakhini Z. (2009). GOrilla: A tool for discovery and visualization of enriched GO terms in ranked gene lists. BMC Bioinf. 10, 48. doi: 10.1186/1471-2105-10-48 PMC2644678 19192299

[B22] Edgar R. Domrachev M. Lash A. E. (2002). Gene Expression Omnibus: NCBI gene expression and hybridization array data repository. Nucleic Acids Res. 30, 207–210. doi: 10.1093/nar/30.1.207 11752295 PMC99122

[B23] Farooq M. Wahid A. Kobayashi N. Fujita D. Basra S. M. A. (2009). Plant drought stress: effects, mechanisms and management. Agron. Sustain. Dev. 29, 185–212. doi: 10.1051/agro:2008021

[B24] Flexas J. Bota J. Loreto F. Corinic G. Sharkey T. D. (2004). Diffusive and metabolic limitations to photosynthesis under drought and salinity in C3 plants. Plant Biol. 6, 269–279. doi: 10.1055/s-2004-820867 15143435

[B25] Flutsch S. Nigro A. Conci F. Fajkus J. Thalmann M. Trtílek M.. (2020). Glucose uptake to guard cells via STP transporters provides carbon sources for stomatal opening and plant growth. EMBO Rep. 21, e49719. doi: 10.15252/embr.201949719 32627357 PMC7403697

[B26] Gao X. Liu K. Lu Y. T. (2010). Specific roles of AtEXPA1 in plant growth and stress adaptation. Russian J. Plant Physiol. 57, 241–246. doi: 10.1134/S1021443710020111

[B27] Gigli-Bisceglia N. Engelsdorf T. Hamann T. (2020). Plant cell wall integrity maintenance in model plants and crop species-relevant cell wall components and underlying guiding principles. Cell. Mol. Life Sci. 77, 2049–2077. doi: 10.1007/s00018-019-03388-8 31781810 PMC7256069

[B28] Guo W. Zhao J. Li X. Qin L. Yan X. Liao H. (2011). A soybean β-*EXPANSIN* gene GmEXPB2 intrinsically involved in root system architecture responses to abiotic stresses. Plant J. 66, 541–552. doi: 10.1111/j.1365-313X.2011.04511.x 21261763

[B29] Hamann T. (2015). The plant cell wall integrity maintenance mechanism concepts for organization and mode of action. Plant Cell Physiol. 56, 215–223. doi: 10.1093/pcp/pcu164 25416836

[B30] Hao Y. Y. Chu L. W. He X. J. Zhao S. T. Tang F. (2024). *PagEXPA1* combines with *PagCDKB2;1* to regulate plant growth and the elongation of fibers in *Populus alba x Populus glandulosa* . Intern. J. Biol. Macromol. 268, 131559. doi: 10.1016/j.ijbiomac.2024 38631576

[B31] Hao Z. Qian X. Xiao X. Huabo L. Junkai Z. Jichen X. (2017). Transgenic tobacco plants expressing grass *AstEXPA1* gene show improved performance to several stresses. Plant Biotechnol. Rep. 11, 331–337. doi: 10.1007/s11816-017-0454-7

[B32] Hepworth C. Doheny-Adams T. Hunt L. Cameron D. D. Gray J. E. (2015). Manipulating stomatal density enhances drought tolerance without deleterious effect on nutrient uptake. New Phytol. 208, 336–341. doi: 10.1111/nph.13598 26268722 PMC4973681

[B33] Huang G. T. Ma S. L. Bai L. P. Zhang L. Ma H. Jia P.. (2012). Signal transduction during cold, salt, and drought stresses in plants. Mol. Biol. Rep. 39, 969–987. doi: 10.1007/s11033-011-0823-1 21573796

[B34] Kok B. O. Altunoglu Y. C. Oncul A. B. Karaci A. Baloglu M. C. (2023). Expansin gene family database: A comprehensive bioinformatics resource for plant expansin multigene family. J. Bioinform. Compl. Biol. 21 (3), 2350015. doi: 10.1142/S0219720023500154 37382165

[B35] Kuluev B. R. Knyazev A. B. Lebedev Y. P. Chemeris A. V. (2012). Morphological and physiological characteristics of transgenic tobacco plants expressing expansin genes: *AtEXP10* from *Arabidopsis* and *PnEXPA1* from poplar. Russ. J. Plant Physiol. 59, 97–104. doi: 10.1134/S1021443712010128

[B36] Kwon Y. R. Lee H. J. Kim K. H. Hong S. W. Lee S. J. Lee H. (2008). Ectopic expression of Expansin3 or Expansin b 1 causes enhanced hormone and salt stress sensitivity in *Arabidopsis* . Biotechnol. Lett. 30, 1281–1288. doi: 10.1007/s10529-008-9678-5 18317696

[B37] Lee D. K. Ahn J. H. Song S. K. Choi Y. D. Lee J. S. (2003). Expression of an expansin gene is correlated with root elongation in soybean. Plant Physiol. 131, 985–997. doi: 10.1104/pp.009902 12644651 PMC166864

[B38] Lee Y. Kende H. Cho H. T. (2001). *EXPANSIN*s: ever-expanding numbers and functions. Curr. Opin. Plant Biol. 4, 527–532. doi: 10.1016/S1369-5266(00)00211-9 11641069

[B39] Li Y. Jones L. McQueen-Mason S. (2003). *EXPANSIN*s and cell growth. Curr. Opin. Plant Biol. 6, 603–610. doi: 10.1016/j.pbi.2003.09.003 14611960

[B40] Li J. F. Liu L. T. Wang L. J. Rao I. M. Wang Z. Y. Chen Z. J. (2024). *AcEXPA1*, an α-expansin gene, participates in the aluminum tolerance of carpetgrass (*Axonopus compressus*) through root growth regulation. Plant Cell Rep. 43, 159. doi: 10.1007/s00299-024-03243-6 38822842

[B41] Li F. Xing S. Guo Q. Zhao M. Zhang J. Gao Q.. (2011). Drought tolerance through over-expression of the expansin gene *TaEXPB23* in transgenic tobacco. J. Plant Physiol. 168, 960–966. doi: 10.1016/j.jplph.2010.11.023 21316798

[B42] Liu X. Q. Cai Y. P. Yao W. W. Chen L. Hou W. S. (2024). The soybean *NUCLEAR FACTOR-Y C4* and *α-EXPANSIN 7* module influences phosphorus uptake by regulating root morphology. Plant Physiol. 197 (1), kiae478. doi: 10.1093/plphys/kiae478 39250753

[B43] Liu Y. Zhang L. Hao W. Zhang L. Liu Y. Chen L. (2019). Expression of two α−type expansins from *Ammopiptanthus nanus* in *Arabidopsis thaliana* enhance tolerance to cold and drought stresses. Int. J. Mol. Sci. 20, 5255. doi: 10.3390/ijms20215255 31652768 PMC6862469

[B44] Lohoff C. Buchholz P. C. F. Le-Roes-Hill M. Pleiss J. (2020). The Expansin engineering database: A navigation and classification tool for expansins and homologues. Proteins 89, 149–162. doi: 10.1002/prot.26001 32862462

[B45] Lu P. Kang M. Jiang X. Dai F. Gao J. ZhangG C. (2013). *RhEXPA4*, a rose *EXPANSIN* gene, modulates leaf growth and confers drought and salt tolerance to *A. thaliana* . Planta 237, 1547–1559. doi: 10.1007/s00425-013-1867-3 23503758

[B46] Marowa P. Ding A. Kong Y. (2016). Expansins: Roles in plant growth and potential applications in crop improvement. Plant Cell Rep. 35, 949–965. doi: 10.1007/s00299-016-1948-4 26888755 PMC4833835

[B47] Marowa P. Dinga A. Xua Z. Konga Y. (2020). Overexpression of *NtEXPA11* modulates plant growth and development and enhances stress tolerance in tobacco. Plant Physiol. Biochem. 151, 477–485. doi: 10.1016/j.plaphy.2020.03.033 32299052

[B48] McQueen-Mason S. J. Cosgrove D. J. (1995). *EXPANSIN* mode of action on cell walls. Analysis of wall hydrolysis, stress relaxation, and binding. Plant Physiol. 107, 87–100. doi: 10.1104/pp.107.1.87 11536663 PMC161171

[B49] Mira W. Heinz O. Goncalvez A. Crema L. Vicentini R. Cardoso S.. (2024). *SacEXP32* sugarcane expansin gene expression increases cell size and improves biomass digestibility. J. Plant Biochem. Biotechnol. 33, 313–325. doi: 10.1007/s13562-024-00891-3

[B50] Murashige T. Skoog F. (1962). A revised medium for rapid growth and bioassays with tobacco tissue. Physiol. Plant. 15, 493–497. doi: 10.1111/j.1399-3054.1962.tb08052.x

[B51] Narayan J. A. Chakravarthi M. Nerkar G. Manoj V. M. Dharshini S. Subramonian N.. (2021). Overexpression of expansin *EaEXPA1*, a cell wall loosening protein enhances drought tolerance in sugarcane. Ind. Crops Prod. 159, 113035. doi: 10.1016/j.indcrop.2020.113035

[B52] Narayan J. A. Dharshini S. Manoj V. M. Padmanabhan T. S. S. Kadirvelu K. Suresha G. S.. (2019). Isolation and characterization of water-deficit stress-responsive α-expansin 1 (*EXPA1*) gene from *Saccharum complex.* 3. Biotech 9, 186. doi: 10.1007/s13205-019-1719-3 PMC6478779 31065486

[B53] Novakovic L. Guo T. Bacic A. Sampathkumar A. Johnson K. L. (2018). Hitting the wall—Sensing and signaling pathways involved in plant cell wall remodeling in response to abiotic stress. Plants 7, 89. doi: 10.3390/plants7040089 30360552 PMC6313904

[B54] Poeschl Y. Möller B. Müller L. Bürstenbinder K. (2020). User-friendly assessment of pavement cell shape features with PaCeQuant: Novel functions and tools. Methods Cell Biol. 160, 349–363. doi: 10.1016/bs.mcb.2020.04.010 32896327

[B55] Ramakrishna P. Ruiz Duarte P. Rance G. A. Schubert M. Vordermaier V. Dai Vue L.. (2019). EXPANSIN A1-mediated radial swelling of pericycle cells positions anticlinal cell divisions during lateral root initiation. Proc. Natl. Acad. Sci. 116, 8597–8602. doi: 10.1073/pnas.1820882116 30944225 PMC6486723

[B56] Ren Y. Chen Y. An J. Zhao Z. Zhang Z. Wang Y.. (2018). Wheat expansin gene *TaEXPA2* is involved in conferring plant tolerance to cd toxicity. Plant Sci. 270, 245–256. doi: 10.1016/j.plantsci.2018.02.022 29576078

[B57] Richter J. Watson J. M. Stasnik P. Borowska M. Neuhold J. Berger M.. (2018). Multiplex mutagenesis of four clustered *CrRLK1L* with CRISPR/Cas9 exposes their growth regulatory roles in response to metal ions. Sci. Rep. 8, 12182. doi: 10.1038/s41598-018-30711-31 30111865 PMC6093868

[B58] Rui Y. Dinneny J. R. (2020). A wall with integrity: Surveillance and maintenance of the plant cell wall under stress. New Phytol. 225, 1428–1439. doi: 10.1111/nph.v225.4 31486535

[B59] Salehi-Lisar S. Y. Bakhshayeshan-Agdam H. (2016). “Drought stress in plants: Causes, consequences, and tolerance,” in Drought Stress Tolerance in Plants, vol. 1 . Eds. Hossain M. Wani S. Bhattacharjee S. Burritt D. Tran L. S. (Switzerland: Springer International Publishing), 1–16.

[B60] Samalova M. Gahurova E. Hejatko J. (2022). Expansin-mediated developmental and adaptive responses: a matter of cell wall biomechanics. Quantitative. Plant Biol. 3, e11. doi: 10.1017/qpb.2022.6 PMC10095946 37077967

[B61] Samalova M. Kirchhelle C. Moore I. (2019). Universal methods for transgene induction using the dexamethasone-inducible transcription activation system pOp6/LhGR in *Arabidopsis* and other plant species. Curr. Protoc. Plant Biol. 4, e20089. doi: 10.1002/cppb.20089 30860661

[B62] Samalova M. Melnikava A. Elsayad K. Peaucelle A. Gahurova E. Gumulec J.. (2024). Hormone-regulated expansins: Expression, localization, and cell wall biomechanics in *Arabidopsis* root growth. Plant Physiol. 194, 209–228. doi: 10.1093/plphys/kiad228 PMC10762514 37073485

[B63] Sampedro J. Cosgrove D. J. (2005). The *EXPANSIN* superfamily. Genome Biol. 6, 242. doi: 10.1186/gb-2005-6-12-242 16356276 PMC1414085

[B64] Sanchez-Munoz R. Depaepe D. Samalova M. Hejatko J. Zaplana I. van der Straeten D. (2024). The molecular core of transcriptome responses to abiotic stress in plants: a machine learning-driven meta-analysis. BioRxiv. Preprint. doi: 10.1101/2024.01.24.576978 PMC12098884 40404615

[B65] Schipper O. Schaefer D. Reski R. Flemin A. (2002). Expansins in the bryophyte *Physcomitrella patens* . Plant Mol. Biol. 50, 789–802. doi: 10.1023/A:1019907207433 12374308

[B66] Shao H. B. Chu L. Y. Jaleel C. A. Zhao C. X. (2008a). Water-deficit stress-induced anatomical changes in higher plants. Comptes. Rendus. Biol. 331, 215–225. 95. doi: 10.1016/j.crvi.2008.01.002 18280987

[B67] Shao H. B. Song W. Y. Chu L. Y. (2008b). Advances of calcium signals involved in plant anti-drought. Comptes. Rendus. Biol. 331, 587–596. doi: 10.1016/j.crvi.2008.03.012 18606388

[B68] Somyong S. Phetchawang P. Bihi A. K. Sonthirod C. Kongkachana W. Sangsrakru D.. (2022). A SNP variation in an expansin *EgExp4* gene affects height in oil palm. PEERJ 10, e13046. doi: 10.7717/peerj.13046 35313525 PMC8934041

[B69] Su G. Q. Lin Y. F. Wang C. F. Lu J. Liu Z. M. He Z. R.. (2024). Expansin SlExp1 and endoglucanase SlCel2 synergistically promote fruit softening and cell wall disassembly in tomato. Plant Cell 36, 709–726. doi: 10.1093/plcell/koad291 38000892 PMC10896287

[B70] Tenhaken R. (2015). Cell wall remodeling under abiotic stress. Front. Plant Sci. 5, 771. doi: 10.3389/fpls.2014.00771 25709610 PMC4285730

[B71] Vaahtera L. Schulz J. Hamann T. (2019). Cell wall integrity maintenance during plant development and interaction with the environment. Nat. Plants 5, 924–932. doi: 10.1038/s41477-019-0502-0 31506641

[B72] Wei P. Chen S. Zhang X. Zhao P. Xiong Y. Wang W.. (2011). An a-expansin, *VfEXPA1*, is involved in regulation of stomatal movement in *Vicia faba* L. Chin. Sci. Bull. 56, 3531–3537. doi: 10.1007/s11434-011-4817-0

[B73] Wolf S. (2022). Cell wall signaling in plant development and defense. Annu. Rev. Plant Biol. 73, 323–353. doi: 10.1146/annurev-arplant-102820-095312 35167757

[B74] Wu Y. Thorne E. T. Sharp R. E. Cosgrove D. J. (2001). Modification of *EXPANSIN* transcript levels in the maize primary root at low water potentials. Plant Physiol. 126, 1471–1479. doi: 10.1104/pp.126.4.1471 11500546 PMC117147

[B75] Yang C. Jiang L. Q. Leng Z. M. Yuan S. Wang Y. Liu G.. (2024). Overexpression of *NtEXPA7* promotes seedling growth and resistance to root-knot nematode in tobacco. Biochem. Biophys. Res. Commun. 720, 150086. doi: 10.1016/j.bbrc.2024.150086 38761478

[B76] Yaqoob A. Bashir S. Rao A. Q. Shahid A. A. (2020). Transformation of α-EXPA1 gene leads to an improved fibre quality in *Gossypium hirsutum* . Plant Breed. 139, 1213–1220. doi: 10.1111/pbr.12878

[B77] Zhang B. Chang L. Sun W. Ullah A. Yang X. (2021). Overexpression of an expansin-like gene, *GhEXLB2* enhanced drought tolerance in cotton. Plant Physiol. Biochem. 162, 468–475. doi: 10.1016/j.plaphy.2021.03.018 33752135

[B78] Zhang X. W. Wang Y. Liu M. Y. Yan P. W. Niu F. Ma F. Y.. (2024). *OsEXPA7* encoding an expansin affects grain size and quality traits in rice (Oryza sativa L.). Rice 17, 36. doi: 10.1186/s12284-024-00715-x 38780864 PMC11116307

[B79] Zhang X. Q. Wei P. C. Xiong Y. M. Yang Y. Chen J. Wang X. C. (2011). Overexpression of the *Arabidopsis* α-expansin gene *AtEXPA1* accelerates stomatal opening by decreasing the volumetric elastic modulus. Plant Cell Rep. 30, 27–36. doi: 10.1007/s00299-010-0937-2 20976459

