# Supplementary figures and images for: Differential expression and localization of expansins in Arabidopsis shoots: implications for cell wall dynamics and drought tolerance
Source: Front Plant Sci. 2025 Feb 10;16:1546819. doi: 10.3389/fpls.2025.1546819 (PMC11847903; doi:10.3389/fpls.2025.1546819)

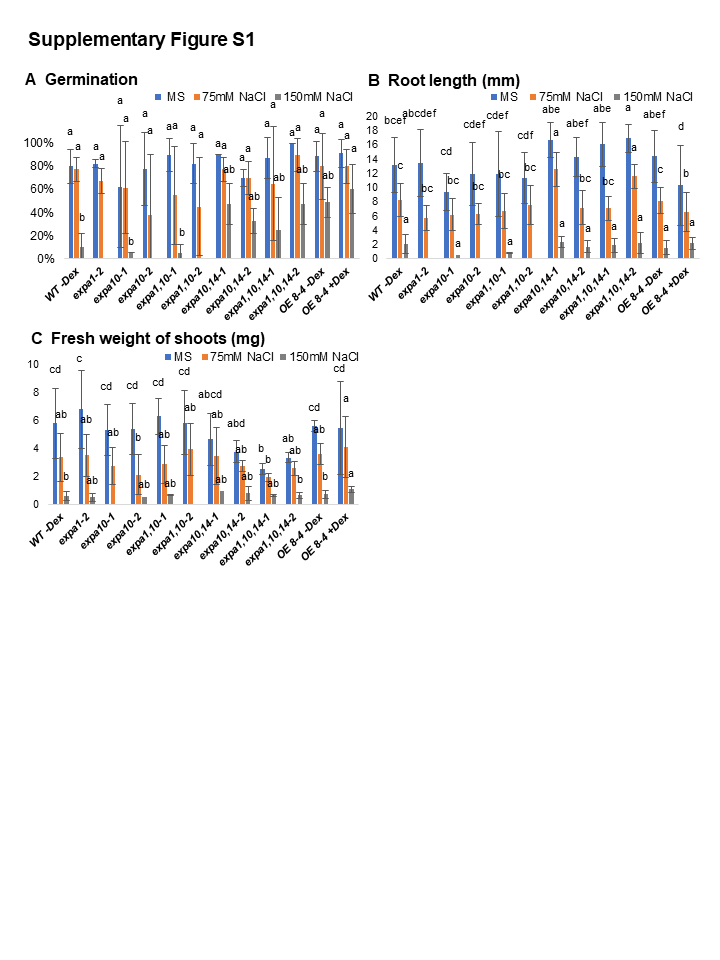

Supplement: Supplementary Figure 1 — Dose-response effect of osmotic stress on Arabidopsis seedlings grown in vitro. Graphs represent (A) germination rate (%), (B) root length (mm), and (C) fresh weight (mg) of shoots of WT, expansin mutants (expa1-2, expa10-1 and -2, expa1,10-1 and -2, expa10,14-1 and -2, expa1,10,14-1 and -2) and EXPA1 OE line (8-4; -Dex and +Dex) grown on MS media supplemented with various concentrations of NaCl (as indicated) for 7 days. Error bars represent S.D., with at least 10 plants per treatment and line analyzed. [file Image1.tif]

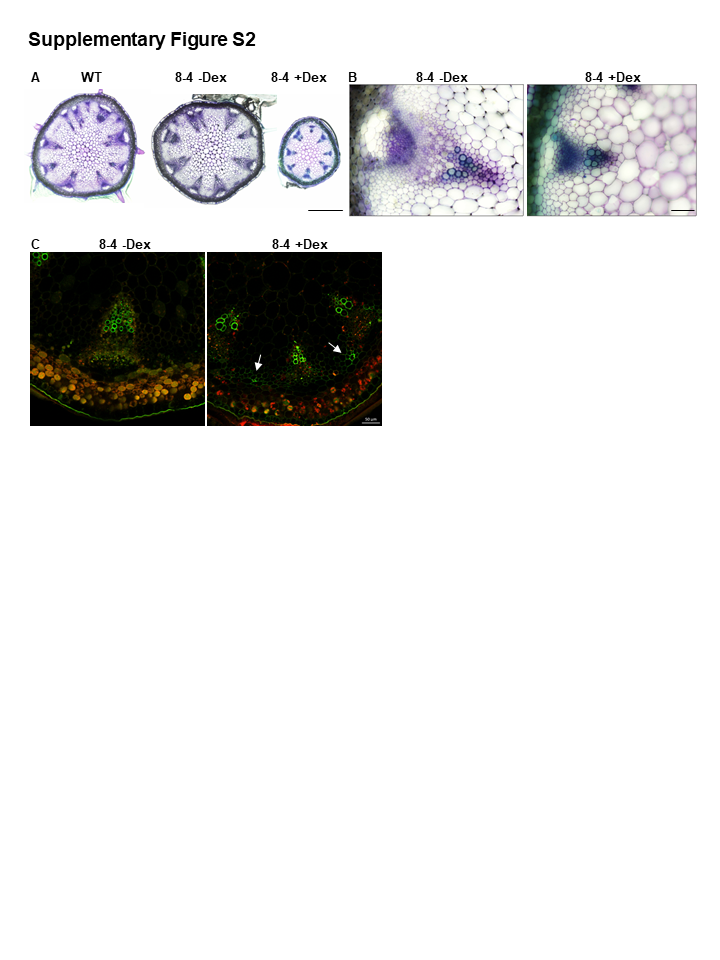

Supplement: Supplementary Figure 2 — EXPA1 overexpression reduces the diameter of Arabidopsis stem. Plants of wild-type (WT) and Dex-inducible EXPA1 OE line 8-4 were grown in soil as described for the control condition experiment and either treated with Dex (+Dex) or left untreated (-Dex) until app. 10-week-old. Inflorescence stem cross-sections were prepared using a vibratome and analyzed as follows: (A) sections stained with toluidine blue visualized using Olympus BX61 microscope, with a detailed view in (B). (C) Sections stained with calcofluor white in green (excitation 458 nm/detection 499-579 nm) imaged alongside autofluorescence in red (excitation 561 nm/detection 588-678 nm) using a Zeiss 880 CLSM. The arrows point at possible onset of precocious secondary CW formation in the interfascicular regions. Scale bars correspond 0.5 mm in (A) and 50 μm in (B, C). [file Image2.tif]

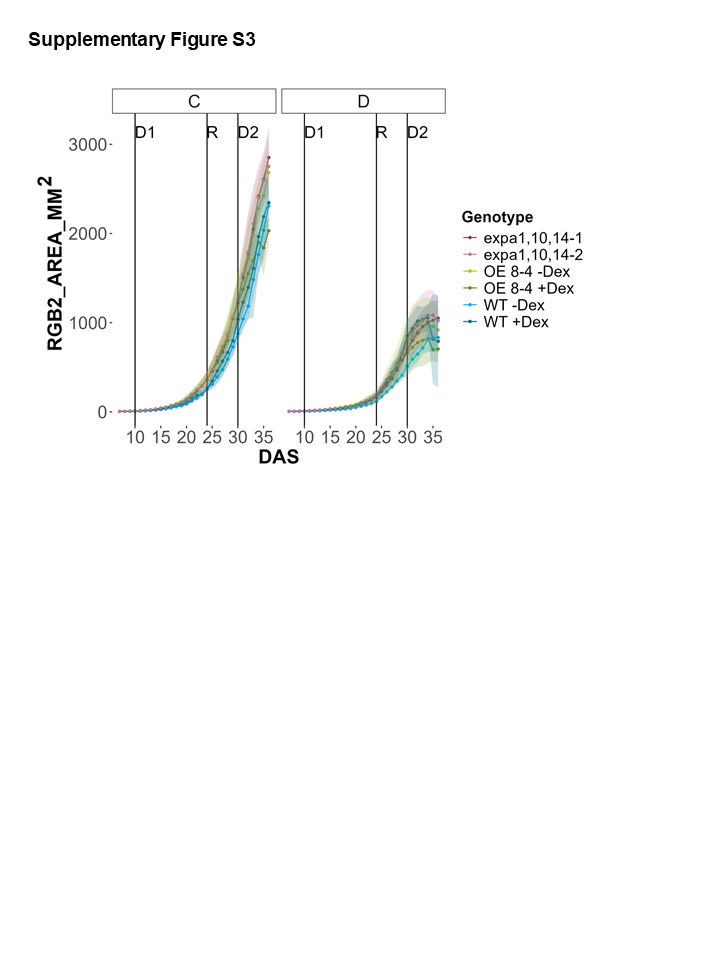

Supplement: Supplementary Figure 3 — Plant area from RGB top view-imaging along DAS. Solid lines represent stress induction phases, where D1 and D2 correspond to the first and second drought stress phases, respectively, and R indicates the recovery phase. The data represent mean values, with shaded areas indicating standard deviation (S.D.). [file Image3.tif]

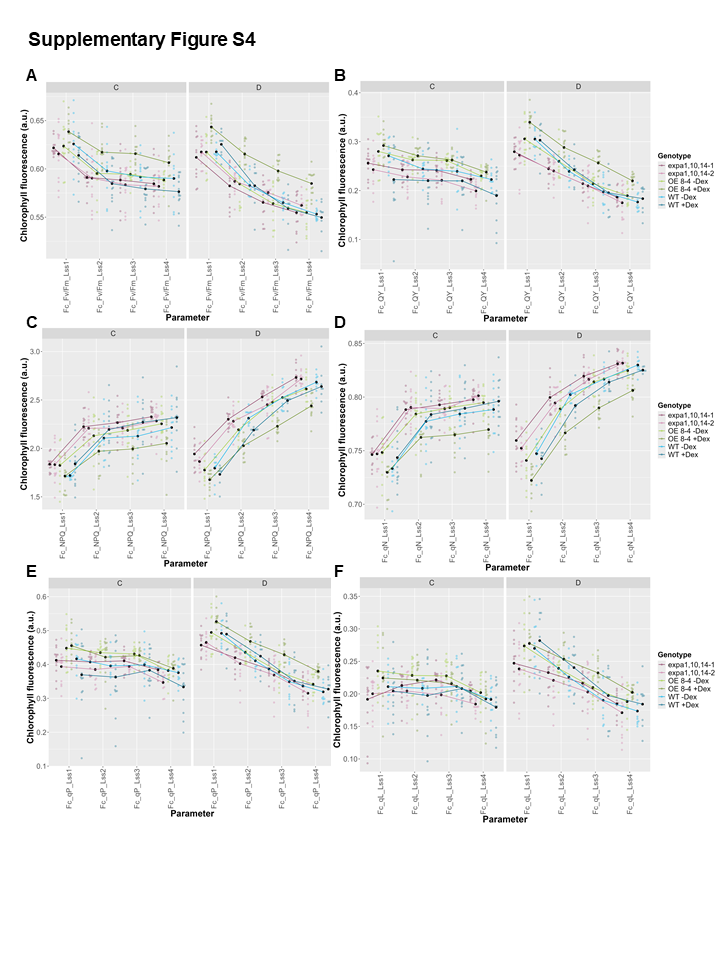

Supplement: Supplementary Figure 4 — Light response curve using kinetic chlorophyll fluorescence imaging. (A) PSII maximum efficiency of light-adapted sample (FV/FM_Lss), (B) PSII quantum yield at light steady-state indicating operating efficiency of PSII (QY_Lss), (C) Non-photochemical quenching at steady-state (NPQ_Lss), (D) Coefficient of non-photochemical quenching in light steady state (qN_Lss), (E) Coefficient of photochemical quenching in steady-state (qP_Lss) and (F) Fraction of PSII centers that are ‘open’ in steady state (qL_Lss). Data were collected under four light levels at 13 DAS. [file Image4.tif]

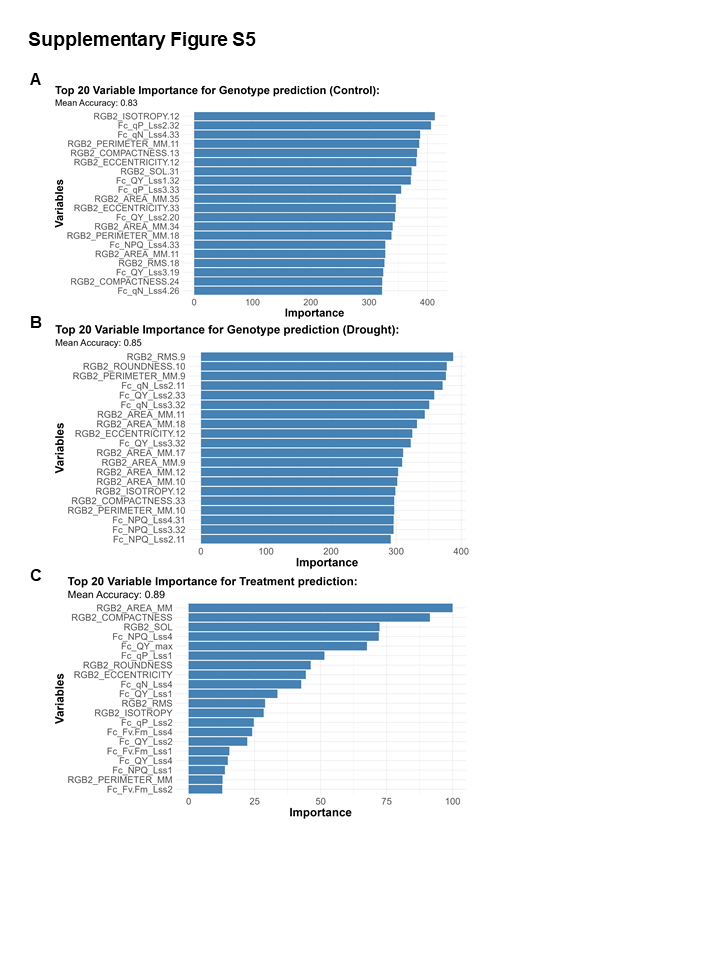

Supplement: Supplementary Figure 5 — Variable importance of traits identified by the Random Forest model across all time points. Morphological and physiological traits that contributed the most to discriminating between the genotypes under (A) control and (B) drought conditions at specific DAS. (C) Key traits of high importance that differentiate between treatments among all lines. The mean accuracy metric indicates the prediction accuracy of the random forest model. [file Image5.tif]
