# Supplementary material for: Differential expression and localization of expansins in Arabidopsis shoots: implications for cell wall dynamics and drought tolerance
Source: Front Plant Sci. 2025 Feb 10;16:1546819. doi: 10.3389/fpls.2025.1546819 (PMC11847903; doi:10.3389/fpls.2025.1546819)
Supplement: Supplementary Table 1 — Trait description extracted from the RGB and chlorophyll fluorescence imaging. [file Table1.docx]

**Supplementary Table S1. Trait description extracted from the RGB and chlorophyll fluorescence imaging.**

[a.u.] arbitrary units

| **RGB 2 (Top view) imaging** | | | |
| --- | --- | --- | --- |
| **Parameter** | | **Description** | |
| Area [mm^2^] | | Total area of visible plant surface. | |
| Perimeter [mm] | | Length of plant perimeter which is the outer boundary of the projected area of the plant. | |
| Roundness [a.u.] | | Index of the rosette shape.  Ratio between area and perimeter of plant surface, or its convex hull, respectively. | |
| Compactness [a.u.] | | Index including petiole length.  Ratio between Area and surface of convex hull enveloping particular plant. More compact plants usually have shorter petioles and larger leaf blades. | |
| Isotropy [a.u.] | | Index indicates the overall symmetry of the plant's structure. Calculated by the construction of polygon from leaf tips around the outermost points and compute its roundness - analogical to plant roundness. | |
| Eccentricity [a.u.] | | Index describing how much the rosette deviates from being circular. The degree of difference between convex hull area and a circle which has its center in the plant centroid. | |
| RMS [a.u.]  (Rotational Mass symmetry) | | Calculated as the ratio between the focus of the ellipse and its major axis length. An alternative approach for evaluating plant shape. | |
| SOL [a.u.]  (Slenderness of Leaves) | | Calculated as the ratio between square of leaf lengths (sum of distances from plant center to the end of leaf blades) and plant area. | |
| Plant Color Segmentation | | Percentage of number of pixels of color on plant surface. The proportion of the plant that falls into color groups pre-selected by the user. | |
| RGR [mm^-2^ d^-1^] | | Relative growth rate calculated according to this formula: (ln (Area)_Time_n+1_) - (ln (Area)_Time_n_) / ((Time_n+1)-(Time_n)) | |
| **Chlorophyll fluorescence imaging** | | | |
| **Parameter** | **Equation** | | **Description** |
| F_0_ | Measured | | Minimum fluorescence in dark-adapted state |
| F_M_ | Measured | | Maximum fluorescence in dark-adapted state |
| F_V_ | F_M_-F_0_ | | Variable fluorescence in dark-adapted state |
| F_M__Lss | Measured | | Steady-state maximum fluorescence in light |
| F_t__Lss | Measured | | Steady-state fluorescence in light |
| F_0__Lss | F_0_/ ((F_V_/F_M_)+(F_0_/F_M__Lss)) | | Steady-state minimum fluorescence in light |
| F_v__Lss | F_M__Lss-F_0__Lss | | Variable fluorescence in light-adapted state |
| F_q__Lss | F_M__Lss-F_t__Lss | | Fluorescence quenching capacity under actinic light |
| QY_max | F_V_ / F_M_ | | Maximum PSII quantum yield in dark-adapted state |
| F_V_/F_M__Lss | F_V__Lss/F_M__Lss | | PSII maximum efficiency of light-adapted sample |
| QY_Lss | F_q__Lss/ F_M__Lss | | Steady-state PSII quantum yield (operating efficiency of PSII; φ_PSII_) |
| NPQ_Lss | (F_M_ - F_M__Lss)/F_M__Lss | | steady-state non-photochemical quenching |
| qN_Lss | 1-(F_v__Lss/F_v_) | | Coefﬁcient of non-photochemical quenching in steady state |
| qP _Lss | F_q__Lss/F_V__Lss | | Coefficient of photochemical quenching in steady-state |
| qL_Lss | (F_q__Lss/F_V__Lss)*(F_0__Lss/F_t__Lss) | | Fraction of PSII centers that are ‘open’ in steady state |
