# Supplementary material for: Differential expression and localization of expansins in Arabidopsis shoots: implications for cell wall dynamics and drought tolerance
Source: Front Plant Sci. 2025 Feb 10;16:1546819. doi: 10.3389/fpls.2025.1546819 (PMC11847903; doi:10.3389/fpls.2025.1546819)
Supplement: Supplementary Table 2 — GO terms significantly enriched among DEGs identified 3 h after Dex-induced EXPA1 overexpression. [file Table2.docx]

**Supplementary Table S2. GO terms significantly enriched among DEGs identified 3 h after Dex-induced *EXPA1* overexpression.**

| [GO:0009828](http://www.godatabase.org/cgi-bin/amigo/go.cgi?query=GO:0009828&view=details)  P-value 4.31E-5  [GO:0042545](http://www.godatabase.org/cgi-bin/amigo/go.cgi?query=GO:0042545&view=details)  P-value 3.34E-4 | **plant-type cell wall loosening**  + cell wall modification | ***EXPA1* - expansin a1**  ***EXPA2* - expansin a2**  ***EXPA6* - expansin a6**  ***EXPA7* - expansin a7 *EXPA12* - expansin a12**  ***EXPA15* - expansin a15**  ***EXPA17* - expansin a17**  ***EXPB1* - expansin b1**  ***EXPB3* - expansin b3**  ***EXLA2* - expansin-like a2 *EXLA3* - expansin-like a3**  *CYP83B1* - cytochrome p450 83b 1 *CYP79B2* - tryptophan n-monooxygenase 1  *CYP79B3* - tryptophan n-hydroxylase 2 *CYP81F2* - cytochrome p450 81f 2  *GH9B1* - endo-1,4-beta-glucanase  *PME3* - pectin methylesterase 3 *AT1G19900* - glyoxal oxidase-related protein  *AT1G48100* - polygalacturonase |
| --- | --- | --- |
| [GO:0009664](http://www.godatabase.org/cgi-bin/amigo/go.cgi?query=GO:0009664&view=details)  P-value 7.83E-5  [GO:0071555](http://www.godatabase.org/cgi-bin/amigo/go.cgi?query=GO:0071555&view=details)  P-value 1.81E-5 | **plant-type cell wall organization**  + cell wall organization | ***EXPA1* - expansin a1**  ***EXPA2* - expansin a2 *EXPA6* - expansin a6**  ***EXPA7* - expansin a7**  ***EXPA12* - expansin-a12 *EXPA15* - expansin a15**  ***EXPA17* - expansin a17 *EXPB1* - expansin b1 *EXPB3* - expansin b3 *EXLA2* - expansin-like a2**  ***EXLA3* - expansin-like a3**  ***ER - lrr* receptor-like serine/threonine-protein kinase erecta**  ***MYB87* - myb domain protein 87 *TCH4* - xyloglucan endotransglucosylase/hydrolase 22 *AT1G48100* - polygalacturonase**  ***AT4G08410* - proline-rich extensin-like family protein *AT4G08400* - proline-rich extensin-like family protein *AT5G06630* - proline-rich extensin-like family protein *AT5G35190* - proline-rich extensin-like family protein**  *CSLC12* - cellulose-synthase-like c12 *CYP83B1* - cytochrome p450 83b1  *CYP79B2* - tryptophan n-monooxygenase 1  *CYP79B3* - tryptophan n-hydroxylase 2  *CYP81F2* - cytochrome p450 81f2 *GH9B1* - endo-1,4-beta-glucanase  *PME3* - pectin methylesterase 3 |
| [GO:0071669](http://www.godatabase.org/cgi-bin/amigo/go.cgi?query=GO:0071669&view=details) P-value 1.28E-4  [GO:0071554](http://www.godatabase.org/cgi-bin/amigo/go.cgi?query=GO:0071554&view=details)  P-value 7.71E-7 | **plant-type cell wall organization or biogenesis**  + cell wall organization or biogenesis | ***EXPA1* - expansin a1 *EXPA2* - expansin a2**  ***EXPA6* - expansin a6 *EXPA7* - expansin a7**  ***EXPA12* - expansin a12**  ***EXPA15* - expansin a15 *EXPA17* - expansin a17 *EXPB1* - expansin b1 *EXPB3* - expansin b3**  ***EXLA2* - expansin-like a2 *EXLA3* - expansin-like a3 *CSLD5* - cellulose synthase-like protein d5 *CSLE1* - cellulose synthase-like protein e1 *CSLG1* - cellulose synthase-like protein g1**  ***CSLG3* - cellulose synthase-like protein g3 *DWF1* - delta(24)-sterol reductase  *ER - lrr* receptor-like serine/threonine-protein kinase erecta**  ***FLA11* - fasciclin-like arabinogalactan protein 11 *MYB87* - myb domain protein 87 *RHS13* - protein root hair specific 13 *TBL15* - trichome birefringence-like 15 protein**  ***TCH4* - xyloglucan endotransglucosylase/hydrolase 22 *XTH25* - xyloglucan endotransglucosylase/hydrolase 25 *AT1G48100* - polygalacturonase *AT1G71690* - hypothetical protein**  ***AT4G08400* - proline-rich extensin-like family protein  *AT4G08410* - proline-rich extensin-like family protein *AT5G06630* - proline-rich extensin-like family protein *AT5G35190* - proline-rich extensin-like family protein**  *CSLC12* - cellulose synthase-like protein c12 *CYP79B2* - tryptophan n-monooxygenase 1 *CYP79B3* - tryptophan n-hydroxylase 2 *CYP81F2* - cytochrome p450 81f 2 *CYP83B1* - cytochrome p450 83b1 *GH9B1* - endo-1,4-beta-glucanase  *PME3* - pectin methylesterase 3  *RHS8* - xyloglucan-specific galacturonosyltransferase  *XTH12* - xyloglucan endotransglucosylase/hydrolase 12 *XTH15* - xyloglucan endotransglucosylase/hydrolase 15 *XTH16* - xyloglucan endotransglucosylase/hydrolase 16 *XTH17* - xyloglucan endotransglucosylase/hydrolase 17  *XTH18* - xyloglucan endotransglucosylase/hydrolase 18 *XTH19* - xyloglucan endotransglucosylase/hydrolase 19 *XTR6* - xyloglucan endotransglucosylase/hydrolase 23 *XTR8* - xyloglucan endotransglucosylase/hydrolase *AT1G19900* - glyoxal oxidase-related protein |
